# Using an Integrated Framework to Investigate the Facilitators and Barriers of Health Information Technology Implementation in Noncommunicable Disease Management: Systematic Review

**DOI:** 10.2196/37338

**Published:** 2022-07-20

**Authors:** Meekang Sung, Jinyu He, Qi Zhou, Yaolong Chen, John S Ji, Haotian Chen, Zhihui Li

**Affiliations:** 1 College of Pharmacy Seoul National University Seoul Republic of Korea; 2 Vanke School of Public Health Tsinghua University Beijing China; 3 Evidence-Based Medicine Center School of Basic Medical Sciences Lanzhou University Lanzhou China; 4 Institute for Healthy China Tsinghua Universtiy Beijing China

**Keywords:** health information technology, noncommunicable disease management, chronic disease management, systematic review, implementation science

## Abstract

**Background:**

Noncommunicable disease (NCD) management is critical for reducing attributable health burdens. Although health information technology (HIT) is a crucial strategy to improve chronic disease management, many health care systems have failed in implementing HIT. There has been a lack of research on the implementation process of HIT for chronic disease management.

**Objective:**

We aimed to identify the barriers and facilitators of HIT implementation, analyze how these factors influence the implementation process, and identify key areas for future action. We will develop a framework for understanding implementation determinants to synthesize available evidence.

**Methods:**

We conducted a systematic review to understand the barriers and facilitators of the implementation process. We searched MEDLINE, Cochrane, Embase, Scopus, and CINAHL for studies published between database inception and May 5, 2022. Original studies involving HIT-related interventions for NCD management published in peer-reviewed journals were included. Studies that did not discuss relevant outcome measures or did not have direct contact with or observation of stakeholders were excluded. The analysis was conducted in 2 parts. In part 1, we analyzed how the intrinsic attributes of HIT interventions affect the successfulness of implementation by using the intervention domain of the Consolidated Framework for Implementation Research (CFIR). In part 2, we focused on the extrinsic factors of HIT using an integrated framework, which was developed based on the CFIR and the levels of change framework by Ferlie and Shortell.

**Results:**

We identified 51 papers with qualitative, mixed-method, and cross-sectional methodologies. Included studies were heterogeneous regarding disease populations and HIT interventions. In part 1, having a relative advantage over existing health care systems was the most prominent intrinsic facilitator (eg, convenience, improvement in quality of care, and increase in access). Poor usability was the most noted intrinsic barrier of HIT. In part 2, we mapped the various factors of implementation to the integrated framework (the coordinates are shown as *level of change-CFIR*). The key barriers to the extrinsic factors of HIT included health literacy and lack of digital skills (*individual-characteristics of individuals*). The key facilitators included physicians’ suggestions, cooperation (*interpersonal-process*), integration into a workflow, and adequate management of data (*organizational-inner setting*). The importance of health data security was identified. Self-efficacy issues of patients and organizational readiness for implementation were highlighted.

**Conclusions:**

Internal factors of HIT and external human factors of implementation interplay in HIT implementation for chronic disease management. Strategies for improvement include ensuring HIT has a relative advantage over existing health care; tackling usability issues; and addressing underlying socioeconomic, interpersonal, and organizational conditions. Further research should focus on studying various stakeholders, such as service providers and administrative workforces; various disease populations, such as those with obesity and mental diseases; and various countries, including low- and middle-income countries.

## Introduction

### Background

Noncommunicable diseases (NCDs) are the number one cause of death and disability in the world [1]. According to World Health Organization (WHO) estimates, NCDs caused around 1.6 million disease-adjusted life years worldwide in 2019, accounting for 62% of the total disease-adjusted life years [2]. To lessen the impact of NCDs on individuals and the society, investing in better management is critical [3]. However, effective management of NCDs has many challenges, including fragmented health systems, difficulties in information exchange, and a lack of interoperable clinical information systems [4].

Health information technology (HIT) has been highlighted to overcome these barriers. HIT refers to the electronic system used to store, share, and analyze health information. This includes, but is not limited to, electronic health records (EHRs), personal health records, and electronic prescribing [5]. HIT could improve the quality of care by reducing paperwork, reducing medical errors, minimizing repetitive medical tests, enabling the collaboration of medical professionals over long distances, and reducing the cost of treatment of chronically ill patients [6]. In addition, HIT can increase patients’ empowerment by helping them develop self-awareness of NCDs [7,8].

Various health care systems have implemented HIT. In 2017, 94% of hospitals in the United States were using EHR systems for managing clinical data [9]. However, many low- and middle-income countries (LMICs) are not quite finished with adapting HIT [10]. For example, EHR systems are not properly used in more than 50% of developing countries [11,12]. This failure is due to resistance and opposition to changing to electronic systems [13], lack of organizational readiness [14], or lack of funding and lack of technical and computer skills of personnel [15]. Developed countries are also heading toward the adaptation of next-generation HITs [16], such as personal health records, patient-centered care, multi-disciplinary care, health information exchange, and integration of artificial intelligence into the health care system. In any case, implementing HIT is challenging, and thus, it is critical to analyze the barriers and facilitators of HIT implementation.

### Prior Work

Implementation of HIT is affected by both the inherent characteristics of HIT (eg, the novelty of the technology and advantages HIT gives to users) and the external factors of HIT (eg, perceptions and behaviors related stakeholders have about implementing new technology). Some studies explored the challenges in a general context, where design and usability issues were mentioned [17-19]. These studies have limitations in understanding the perspectives of various stakeholders. Other previous research concentrated on a specific topic, such as diabetes management [20-25] or one type of HIT (eg, patient web portal) [22], which is insufficient for understanding HIT implementation in a more general setting. Frameworks have helped understand the implementation processes of various topics. For example, Webb et al [8] integrated the level theory by Ferlie and Shortell to understand perinatal mental health care, and Esponda et al used the Consolidated Framework for Implementation Research (CFIR) [26] to analyze mental health implementation [27]. However, determinant frameworks have been used scarcely in understanding HIT implementation. The existing frameworks also have limitations in differentiating between whether a factor is an intrinsic characteristic of HIT or a human factor related to the stakeholders.

### The Goal of This Study

Therefore, our objective was to tackle the research gap regarding the implementation of HIT for chronic disease management. We specifically aimed to identify the barriers and facilitators, analyze how these factors influence the HIT implementation process, and identify key areas for future action. We will develop a framework for understanding implementation determinants to synthesize available evidence.

## Methods

### Search Strategy and Selection Criteria

In this systematic review, literature searches and study selection followed the Preferred Reporting Items for Systematic Reviews and Meta-Analyses guidelines [28] (Multimedia Appendix 1). As the review did not evaluate a direct health-related outcome, it did not meet the criteria for registration of the protocol with PROSPERO. The author MS searched the MEDLINE, Cochrane, Embase, CINAHL, and Scopus databases for research articles published between database inception and May 5, 2022.

Boolean operators were used to combine relevant search terms related to NCDs (eg, “noncommunicable diseases,” “chronic diseases,” “diabetes,” and “hypertension”), HIT (eg, “health information technology,” “electronic health records,” “personal health records,” and “electronic prescribing”), and implementation outcomes (eg, “barrier” and “facilitator”). Based on the definition of HIT [5], search phrases for HIT also included a wide range of HIT-related literature.

The search syntax was devised and written by MS and reviewed by ZL. The full search syntax can be found in Multimedia Appendix 2. The initial search was completed on August 11, 2021. Forward and backward searches of included studies were completed by October 31, 2021. The supplementary search was completed by May 5, 2022.

Studies were eligible if they involved HIT-related interventions (eg, EHRs, personal health records, and electronic prescribing), involved interventions that were used for NCD management, and examined implementation outcomes (ie, barriers or facilitators). Studies were included if they were published in peer-reviewed academic journals and had direct contact with or direct observation of different stakeholders, such as patients, the public (consumers), companies, and health professionals. The articles included were required to have full text available and be written in English.

Studies were excluded if they were not related to chronic disease management, did not implement HIT-related interventions (eg, studies that concentrated on digital health interventions that were not related to HIT), had an outcome that was not focused on implementation, or did not discuss facilitators and barriers (eg, studies that reviewed the effectiveness of HIT).

### Study Selection

Search results were imported into EndNote 20 (Clarivate). After removing duplicates, MS and JH independently double-screened all titles and abstracts. The interrater reliability between the first and second screeners was 58% in the first screening. Both authors discussed all disagreements and were able to agree on all selections of papers (κ=100%). The full texts of the included papers were then assessed for eligibility by MS and JH. The interrater reliability (κ) was 71% in the initial selection of full-text papers. Both authors discussed all disagreements and came to an agreement on all included studies. If necessary, a third author (ZL) mediated agreement.

### Data Collection and Data Items

Extraction of data on author, year, country, study design, data collection methods, participants, intervention stage, target population, HIT program/intervention, and addressed stakeholders was performed by MS and JH into an Excel spreadsheet (Microsoft Corp). The full texts of the studies were also extracted to NVivo (Release 1.5) software (QSR International), which allows for line-by-line coding. Each paper was read in full, and relevant parts of the text were applied to the relevant code. Data extraction followed the data extraction form (Multimedia Appendix 3), which was guided by the Cochrane Systematic Review for Intervention Data Collection form [29].

### Critical Appraisal of Studies

MS and JH independently conducted quality assessments of the included studies using several appraisal tools based on the type of research. Joanna Briggs Critical Appraisal Tools were used for qualitative research [30], the Mixed Methods Appraisal Tool [31] was used for mixed methods studies, and the Center for Evidence-Based Management Critical Appraisal Checklist was used for cross-sectional studies [32]. Multimedia Appendix 4 explains each quality appraisal method in detail. Each point of the Joanna Briggs Critical Appraisal Tools can be coded into either yes, no, unclear, or not applicable. Each point of the Mixed Methods Appraisal Tool and the Center for Evidence-Based Management Critical Appraisal Checklist can be coded into yes, no, or cannot tell. Where most questions within a domain or a paper were answered with yes, it was rated as having high quality, and where the majority were answered with no, it was rated as having low quality. Medium quality was when there was a mixture of yes and no answers. The note in Multimedia Appendix 5 explains the detailed criteria for high, medium, and low quality for each type of research. Studies were not excluded based on quality to capture as much literature as possible, but low-quality studies were not used to draw conclusions.

### Synthesis of Results

Enhancing Transparency in Reporting the Synthesis of Qualitative Research guidelines were followed (Multimedia Appendix 6) [33]. We used the best-fit framework synthesis approach [34]. First, statements referring to facilitators or barriers of the implementation of HIT-related interventions were extracted line by line. Second, full texts of studies were exported to NVivo for analysis. Statements referring to facilitators or barriers of the implementation of HIT-related interventions were extracted line by line and coded. Third, codes were reread and assigned a descriptive theme based on their content. Once all codes were assigned, various implementation frameworks were assessed for their fit with the existing frameworks (eg, CFIR [26], Reach Effectiveness Adoption Implementation Maintenance [35], socioecological model [36], and levels of change framework by Ferlie and Shortell [37]) to structure themes. The CFIR and the levels of change framework were selected since they best matched the codes and descriptive themes that were derived in this review.

**Figure 1:**
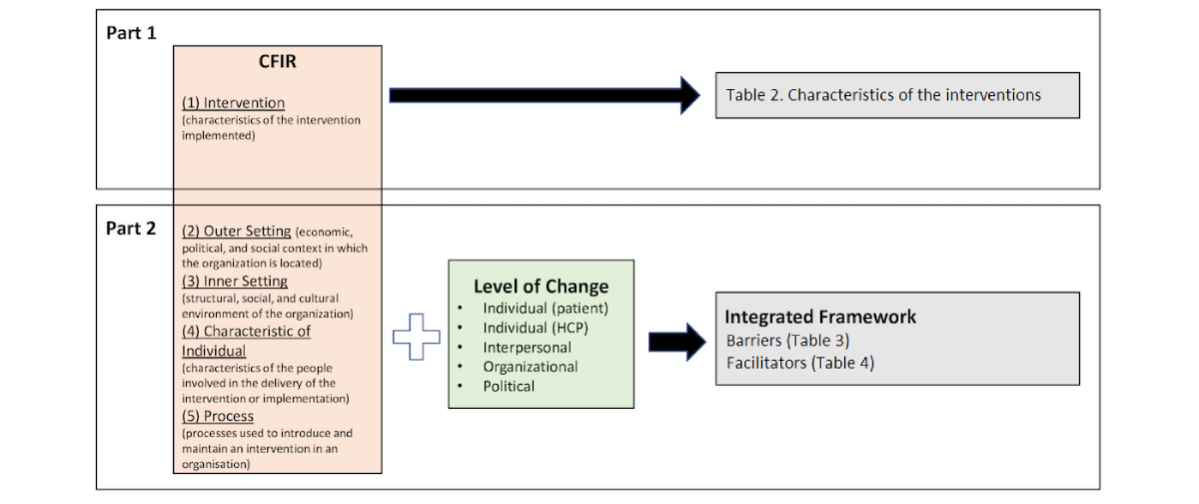
Study design. CFIR: Consolidated Framework for Implementation Research; HCP: health care provider.

Our analysis was conducted in two parts. Figure 1 illustrates the study design. In part 1, we aimed to understand the inherent characteristics of HIT that affect implementation. The intervention domain (“characteristics of the intervention implemented”) of the CFIR was found to fit best and was therefore used. The CFIR, which has been extensively used in research, has a comprehensive categorization of implementation determinants informed by both empirical findings and theory. It is composed of the following 5 domains: (1) intervention, (2) outer setting, (3) inner setting, (4) individuals, and (5) process. The intervention domain is constructed of 8 subconstructs, which help analyze the complex and multi-faceted characteristics of HIT. Among the 8 subconstructs (innovation source, evidence strength and quality, relative advantage, adaptability, trialability, complexity, design quality and usability, cost), “innovation source” and “trialability” did not have matching concepts in our findings and were therefore excluded.

**Figure 2:**
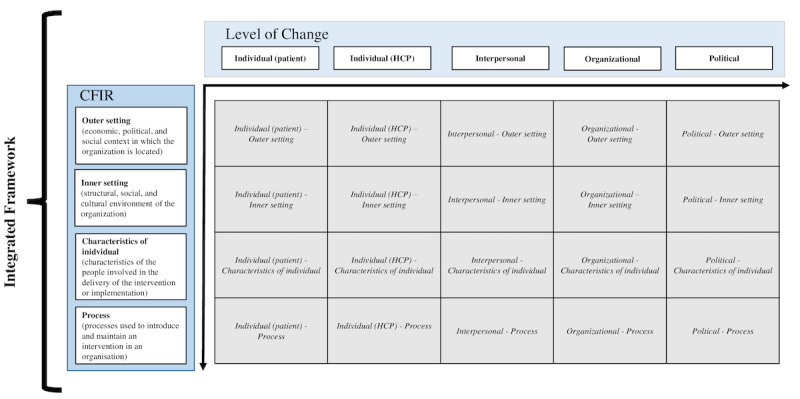
Diagram of the integrated framework. CFIR: Consolidated Framework for Implementation Research; HCP: health care provider.

In part 2, we conducted a stakeholder analysis with the integrated framework. The integrated framework was developed based on the CFIR and the levels of change framework by Ferlie and Shortell, as shown in Figure 2. The latter 4 domains of the CFIR involved various stakeholders and their relations. However, the CFIR is limited in identifying which specific stakeholders are involved with a factor.

The levels of change framework, which is also frequently used in the literature, categorizes factors on the following 4 levels: (1) individual, (2) care team, (3) organizational structure, and (4) the wider environment [37]. This framework compensates for the CFIR because it can identify which stakeholders are involved in a factor. Moreover, it can explain at which level the factors are being affected. However, since it is only constructed of 4 levels, it fails to deliver a specific view and separately categorize disparate factors.

By combining the CFIR and the levels of change framework, we could complement each framework’s weaknesses. We first modified the categories of the levels of change framework as individual factors (patients and health care providers [HCPs]), interpersonal factors, organizational factors, and political factors. Then, we combined the 2 frameworks to develop a novel integrated framework. Themes that could not be explained by the original frameworks were identified and synthesized into the integrated framework. After developing the integrated framework, codes were reread and assigned deductively. Data coding was undertaken with NVivo (Release 1.5) software.

We placed the CFIR constructs on the vertical axis and the level of change categories on the horizontal axis and mapped relevant factors of implementation in matching coordinates (Figure 2). A factor showing “individual” on the horizontal axis and “outer setting” on the vertical axis, for example, acts at the individual level and is related to the outer setting of implementation. The most mentioned *level of change-CFIR* sections are explained in detail in the Results.

This method helps to understand the overall picture because it provides the location (horizontal and vertical) of factors, and the categories are more specified than either the CFIR or the levels of change framework.

## Results

### Study Selection

**Figure 3:**
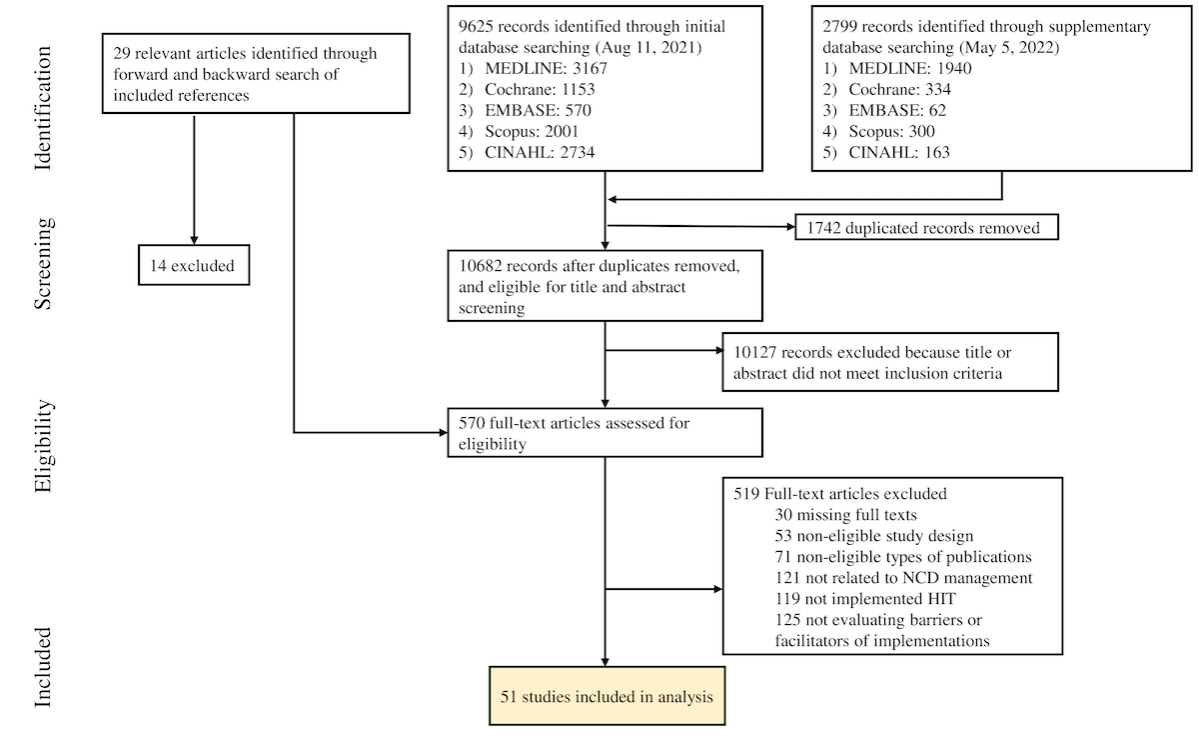
Study selection. HIT: health information technology; NCD: noncommunicable disease.

We identified 12,424 records through database searches (Figure 3). A total of 9625 articles were from the initial search, and additional 2799 articles were added from the supplementary search. After removing duplicates, 10,682 citations were left. During the full-text screening of 555 articles, 29 articles identified by the forward and backward searches of the included references were further screened for eligibility, of which 15 articles were finally added. Of a total of 10,697 articles, 570 were identified as potentially relevant records after screening the titles and abstracts. After full-text screening, 51 studies were included for analysis. Figure 3 describes the number of papers excluded for each exclusion criteria.

Included studies were heterogeneous, with different sample sizes, interventions being implemented, countries of origin, and methodologies. Programs used qualitative, mixed-method, or cross-sectional study designs. A total of 34 studies were qualitative [38-71]. Common qualitative methods for data collection included in-depth interviews and focus groups. The sample sizes of qualitative studies ranged from 18 to 110. Twelve studies used a mixed methods design [72-84]. Common methods for data collection were surveys, questionnaires, or descriptive statistics mixed with qualitative studies. Four studies used quantitative methodology, 3 used cross-sectional survey methodology [85-87], and 1 extracted data from an electronic medical record system [88].

MS and JH independently completed the assessment for the included papers. The appraisal of quality was the same for 37 (73%) of the 51 papers. All disagreements were discussed by SM and HJ, and if necessary, a third author (ZL) mediated agreement. The final appraisal was based on agreed answers. Of the 51 papers, 31 were determined as high quality, 18 as medium quality, and 2 as low quality. The detailed quality evaluation by quality appraisal domains is shown in Multimedia Appendix 5.

Detailed characteristics of the included 51 studies can be found in Multimedia Appendix 7. Most (30/51) of the included studies addressed diabetes [41,42,45,47,49,51-54,56,57,59,60,64,65,​72-77,79-82,85,86,88]. Other target populations addressed were as follows: cancer [40,44,66,68,71,84,87], general primary care [46,50,58,78,83], multiple chronic conditions [38,39,63,70], hypertension [57,73,79], mental health [54,55,60], general health care [61], cardiovascular diseases [69], heart disease [60], hyperlipidemia [79], elderly and disabled [43], and chronic kidney disease [62].

Table 1 presents the characteristics of the included studies by type of HIT intervention, target population, country, and stakeholder. The most reported types of HIT interventions were patient portals [46,49,61,63,74,75,81,83,85,86], electronic health registries [54,57,59,62,66,70,71,84,87,88], clinical decision support systems [50,51,55,64,65,69,73,76,78], personal health records [38,39,42-44,56,58,68,77], integrative care modules [45,60,77,79,82], patient decision aids [47,48], digital education programs [41], self-management programs [80], shared decision-making [53], tailored messages [72], general HIT [67], and other programs [40,52]. Most studies primarily focused on the factors that affect patients or HCPs. Some literature reported other stakeholders, such as information technology employees [61], family [44], caregivers [46,83], vendors [59], care managers [48,61], educators [52], and staff (ie, nurse practitioners and physician assistants) [76].

**Table 1:** Characteristics of the included studies.

Characteristic		Value (N=51), n^a^
**Type of HIT^b^ intervention**		
	Patient portals	10
	Electronic health registries	10
	Personal health records	9
	Clinical decision support systems	9
	Integrative care modules	4
	Patient decision aids	2
	Other HIT-based management	2
	Digital education programs	1
	Self-management programs	1
	Shared decision-making	1
	Tailored messages	1
	General HIT	1
**Target population**		
	Diabetes	30
	Cancer	7
	General primary care	5
	Multiple chronic conditions	4
	Hypertension	3
	Mental health	3
	Heart disease	1
	Hyperlipidemia	1
	Elderly and disabled	1
	Chronic kidney disease	1
**Country**		
	United States	30
	The Netherlands	4
	Canada	4
	Australia	2
	Malaysia	2
	Malawi	2
	United Kingdom	1
	Scotland	1
	Brazil	1
	Finland	1
	Germany	1
	Iran	1
	Uganda	1
**Stakeholder**		
	Patients	37
	Health care providers	27
	Vendors	8
	Staff/clinic manager	5
	Caregivers	2
	Information technology employee	1
	Researcher	1

^a^Number of included studies.

^b^HIT: health information technology.

### Part 1: Inherent Characteristics of HIT Interventions

We coded the inherent characteristics of HIT implementation into barriers and facilitators (Table 2). Detailed definitions and reflective quotes can be found in Multimedia Appendix 8.

Evidence strength and quality was both a facilitator and barrier. A trustworthy knowledge base, such as reliable data sets and recommendations from trusted peers, facilitated HIT use [50]. However, stakeholders would be reluctant in adapting HIT if they did not trust the technology [39,57,75]. For instance, some providers perceived patient-recorded data as unreliable and therefore had a lack of desire to use patient portals [39].

**Table 2:** Inherent characteristics of health information technology interventions as barriers and facilitators.

Characteristic	Barriers	Facilitators
Evidence strength and quality	Unreliability of data [39,57,75] (3 mentions)	Ensuring reliability [25,50,57] (3 mentions)
Relative advantage	Threaten the HCP^a^-patient relationship [49,50], reduce the quality of care [49], unhelpful [49,51,72,76], and provoke negative emotions [38,39] (9 mentions)	Convenience [42,46,49,52,75,81], help HCP-patient communication [46,63-65,70,76,87,89], help monitoring [52], engagement [42,76,82], improve disease management [46,49,56], improve data quality [71,87], improve quality of care [45,46,49,75,80,90], improve awareness [40,49,56,63,76,77], efficiency [63,71,87], increase access [42,45,49,75,77,85,90], perceived usefulness [44,49,51,61,67,69,75,85], and reduce risk of error [87] (54 mentions)
Adaptability	Inapplicability [50,58,76], poor accessibility [49,61,63,77], and interoperability problems [70] (8 mentions)	Flexibility [44,50,51,55,57] and data interoperability [78,90] (7 mentions)
Complexity	Data-related problems (collecting, managing, processing) [49,50,52,60,77,90], technical challenges [41,44,49,63,80,83], and overall complexity [53,80,88] (15 mentions)	None reported (0 mentions)
Design quality and usability^b^	Poor data quality [38,42,51,77,90], poor design [40,44,49,63,77,84], and difficult to use system (eg, password problems, slow speed, functionality) [42,44,46,51,56,58,78,84,90] (20 mentions)	Good data quality [56,72], good design [46], good data visualization [51,52,70,76,77], good content (eg, specific) [72], and ease of use [51,58,61] (12 mentions)
Cost	Cost of implementation [47,48] and cost of the internet [46] (3 mentions)	Technology reduces costs [67,87] (2 mentions)

^a^HCP: health care provider.

^b^The definitions have been modified from the original Consolidated Framework for Implementation Research construct codebook to match the context of this study.

The relative advantage of new technology was mentioned 54 times in the included studies, being the most frequently reported facilitator. The advantages of HIT were increased accessibility [42,45,49,75,77,85,90], 24/7 real-time access [42,49,75,77,85], and being able to acquire up-to-date information at a convenient time [90], which helped patients feel safe [45]. HIT also lessened administrative work for patients and HCPs, such as scheduling and managing appointments [42,75], organizing refill/reauthorization reminders [81], and managing data [52]. Overall, HIT was convenient [49] and helped stakeholders save time [46]. In addition, stakeholders viewed HIT as a valuable instrument for improving the quality of care [45,46,49,75,80,90]. Many examples mentioned how HIT helped improve the quality of face-to-face conversations between HCPs and patients [46,49,75]. It also helped continuous care of medical conditions [45], speed of communication [90], and prevention of medical errors [87,90]. However, HIT was sometimes noted as unhelpful [49,51,72,76] or even provoking negative emotions in the process of managing medical data [38,39]. Adequate adaptability that enables HIT to be tailored to meet various needs was revealed as a facilitator, while inapplicability [50,58,76] and poor accessibility [49,77] acted as barriers. Allowing patient choice over default settings [57], clinician autonomy and flexibility [50,51,55], and up-to-date information contributed to adaptability [44]. “Complexity,” which is the perceived difficulty that hindered the use of the system, was noted several times. Especially, data management problems, such as collecting, managing, and processing data [49,50,52,60,77,90], and frequent technical challenges [41,44,49,63,80,83] were important.

Design quality and usability was the most mentioned barrier (20 times). Inaccurate or incomplete data [38,42,51,69,77,88,90] and poor user interface or inadequate design [49,77] of app/program features were noted (eg, “prompt overload” [40] and “wordiness” [44]). Difficulties in using the system, such as frequent password problems [42,44,46], slow speed of the system [51,56,58,90], and lack of functionality, acted as barriers [51]. On the other hand, good data quality [56,72], good design [46], good data visualization [51,52,70,76,77], good content (eg, specific) [72], and good system usability [51,58] encouraged the use of HIT.

There were differing views regarding the cost of deploying HIT. Several articles regarded the expenses needed for implementing HIT as expensive and burdensome [47,48]. However, other papers suggested that using HIT could save money by lowering health care costs [67,87].

### Part 2: Stakeholder Analysis

We have mapped the barriers (Table 3) and facilitators (Table 4) by the integrated framework. The references of each factor are indicated in Multimedia Appendix 9. Table 5 summarizes the numbers of times the barrier and facilitator codes in the category emerged in the selected papers.

**Table 3:** Stakeholder analysis with the integrated framework for barriers of health information technology implementation.

Barriers		Individual (patient)	Individual (health care professional)	Interpersonal	Organizational	Political
**Outer setting**						
	Needs and resources	Lack of desire (n=4)^a^ and lack of need (n=2)	Lack of desire (n=1) and lack of need (n=1)	N/A^b^	N/A	N/A
	External policy and incentives	N/A	N/A	N/A	N/A	Regulation concerns (n=2), government policies (n=1), and lack of health system support (n=1)
**Inner setting**						
	Structural characteristics	N/A	N/A	N/A	Organizational issues (n=4), unclear responsibilities (n=4), and organizational conflicts (n=1)	N/A
	Networks and communications	N/A	N/A	Lack of connection with peers (n=1) and lack of trust (n=1)	N/A	N/A
	Implementation climate	Feels like work (n=3) and competing priorities (n=2)	Competing priorities (n=3)	N/A	Tension for change (n=1), lack of fit with existing workflow (n=3), competing priorities (n=3), and lack of reimbursement (n=2)	N/A
	Readiness to implementation	Lack of computer or internet (n=5), lack of financial resources (n=1), and lack of training (n=1)	Lack of time (n=7)	Lack of assistance (n=3)	Lack of leadership engagement (n=1), lack of administrative support (n=1), lack of infrastructure and equipment (n=6), lack of financial resources (n=3), lack of workforce (n=3), and increased workload (n=3)	N/A
	Privacy and confidentiality	Privacy concern (n=5)	Privacy concern (n=2)	N/A	N/A	Political regulations (n=1)
**Characteristics of individuals**						
	Knowledge and beliefs	Concerns on diminishing interaction with HCPs^c^ (n=1), high expectations (n=2), lack of knowledge (n=3), and preconceived beliefs (n=3)	Lack of knowledge (n=2), past negative experience (n=2), negative attitude (n=1), resistance toward change (n=2), and concern on patient’s role (n=1)	N/A	N/A	N/A
	Self-efficacy	Health literacy (n=7) and lack of digital skills (n=10)	Lack of digital skills (n=2)	N/A	N/A	N/A
	Other	Cognitive impairment (n=1), financial status (n=1), literacy (n=4), passive attitude (n=1), physical impairment (n=1), and inadequate knowledge of own health (n=2)	Older age (n=2) and poor communication style (n=1)	N/A	N/A	N/A
**Process**						
	Planning	N/A	N/A	N/A	N/A	Lack of long-term plans (n=1)
	Engaging	N/A	Lack of HCP engagement (n=2)	Lack of patient-provider engagement (n=1)	Lack of organizational commitment (n=1)	N/A
	Executing	N/A	N/A	Lack of cooperation (n=1)	N/A	N/A

^a^Throughout the table, “n” refers to the number of times a code emerged in all the selected papers.

^b^N/A: not applicable.

^c^HCP: health care provider.

**Table 4:** Stakeholder analysis with the integrated framework for facilitators of health information technology implementation.

Facilitators		Individual (patient)	Individual (health care professional)	Interpersonal	Organizational	Political
**Outer setting**						
	Needs and resources	Need for management and information (n=1)^a^ and self-motivation (n=2)	Motivation to change (n=1)	N/A^b^	N/A	N/A
	Cosmopolitanism	N/A	N/A	Positive experience of early adopters (n=2)	N/A	N/A
	Peer pressure	N/A	N/A	N/A	Peer pressure (n=1)	N/A
	External policy and incentives	N/A	N/A	N/A	N/A	Laws and regulations (n=1)
**Inner setting**						
	Networks and communications	N/A	N/A	Trusted relationship (n=1) and communication (n=1)	N/A	N/A
	Culture	N/A	N/A	N/A	Innovation-oriented culture (n=1)	N/A
	Implementation climate	Match workflow (n=1)	N/A	N/A	Integration into workflow (n=3)	N/A
	Readiness to implementation	Conducive environment (n=1) and patient education (n=2)	Training (n=3)	N/A	Administrative support (n=2), adequate infrastructure (n=2), adequate financial resources (n=1), and technical support (n=2)	N/A
	Privacy and confidentiality	N/A	N/A	N/A	Adequate management of data (n=3)	N/A
**Characteristics of individuals**						
	Knowledge and beliefs	Adequate knowledge base (n=2)	Positive attitude (n=1)	N/A	N/A	N/A
	Self-efficacy	Adequate health literacy (n=1)	N/A	N/A	N/A	N/A
	Other	N/A	Good communication style (n=1)	N/A	N/A	N/A
**Process**						
	Planning	Strategic implementation process (n=1)	N/A	N/A	N/A	N/A
	Engaging	HCP^c^ engagement (n=1)	Physician’s suggestion (n=5) and family support (n=1)	Identify and nurture champion (n=1)	N/A	N/A
	Executing	N/A	Cooperation (n=3) and patient-provider communication (n=2)	Use pre-existing relationships (n=1)	N/A	N/A
	Reflecting and evaluating	N/A	N/A	Feedback from provider (n=1)	Feedback (n=2) and regular monitoring (n=1)	N/A

^a^Throughout the table, “n” refers to the number of times a code emerged in all the selected papers.

^b^N/A: not applicable.

^c^HCP: health care provider.

**Table 5:** Summary of the stakeholder analysis with the integrated framework.

Variable			Individual (patient), n^a^			Individual (health care professional), n^a^			Interpersonal, n^a^				Organizational, n^a^				Political, n^a^		
			B^b^		F^c^	B	F		B		F		B		F		B		F
**Outer setting**																			
	Needs and resources	6		3		2	1	0		0		0		0		0		0	
	Cosmopolitanism	0		0		0	0	0		2		0		0		0		0	
	Peer pressure	0		0		0	0	0		0		0		1		0		0	
	Eternal policy and interventions	0		0		0	0	0		0		0		0		4		1	
**Inner setting**																			
	Structural characteristics	0		0		0	0	0		2		9		0		0		0	
	Networks and communications	0		0		0	0	2		0		0		0		0		0	
	Culture	0		0		0	0	0		0		0		1		0		0	
	Implementation climate	5		1		3	0	0		0		9		3		0		0	
	Readiness to implementation	7		3		7	3	3		0		17		7		1		0	
	Privacy and confidentiality	5		0		2	0	0		0		0		0		0		0	
**Characteristics of individuals**																			
	Knowledge and beliefs	9		2		8	1	0		0		0		0		0		0	
	Self-efficacy	17		1		2	0	0		0		0		0		0		0	
	Other	10		0		2	1	0		0		0		0		0		0	
**Process**																			
	Planning	0		0		0	0	0		0		1		1		0		0	
	Engaging	0		0		2	1	1		6		1		1		0		0	
	Executing	0		0		0	0	1		6		0		1		0		0	
	Reflecting and evaluating	0		0		0	0	0		0		0		1		0		3	

^a^The number of times the barrier/facilitator codes in the category emerged.

^b^B: barrier.

^c^F: facilitator.

#### Individual (Patient)-Characteristics of Individuals

The individual factors of patients were the most reported barriers and facilitators. Factors associated with the CFIR construct “characteristics of individuals,” particularly self-efficacy issues, were prominent. Many patients did not have sufficient health literacy to understand the content of HIT [38,43,46,48,53,83,86] and were therefore limited in the use of HIT [46] or required assistance [83]. Lack of digital skills for using the computer and the internet challenged HIT use for both patients [41,43,46,56,57,63,66,81,83,85] and HCPs [55,87]. Some studies even revealed the existence of “computer anxiety” [43]. Sometimes patients did not have a computer or an internet connection [46,49,56]. In contrast, having adequate health literacy [46] and knowledge [38,53] acted as facilitators.

Lack of financial resources [56], cognitive impairment [43], literacy [46,61,66,83], passive attitude [76], physical impairment [43], and inadequate knowledge of own health [53] were also barriers. On the other hand, adequate knowledge of the health system and medical data [38,53], and adequate health literacy acted as facilitators [46].

Initial knowledge and beliefs on HIT were also frequently noted. Patients did not know of the existence of HIT [42,81,85] or were not aware of the tool’s functions [51]. Moreover, negative preconceived attitudes toward HIT [38,75,81], such as dislike of electronic communication methods [75,81] and misconceptions about the health care system [38], hindered them from trying something new. They were also worried that a new communication method might diminish the original communication with HCPs [46].

#### Individual (Patient)-Needs and Resources (Outer Setting)

Patients’ lack of wants or needs served as a barrier, whereas their desire for management and drive served as a facilitator. Patients were sometimes disinterested in the self-management of their disease [56,63,66,85] and preferred medical discussions based on personal clinical encounters [79], or already had an alternative method of managing their disease [56]. Medical data tracking was often conceived as effortful and time-consuming [39,56,80]. However, patients were also frequently self-motivated in incorporating HIT into their daily lives [61,65,66].

#### Individual/Organizational/Political-Privacy and Confidentiality (Inner Setting)

The construct “privacy and confidentiality” was added because of the unique characteristic of HIT, that is, it deals with sensitive personal information. Patients mentioned privacy concerns as a barrier to HIT implementation [38,44,46,56,72] (eg, wary about the number of people who might have access to one’s medical records [72]). HCPs were also worried about the possibility of exploiting patient data [59,90]. When sufficient measures were taken to ensure the privacy of medical data, it acted as a facilitator [44,57,72]. The perception of security was increased by features like secure messaging [72], safe storage [57], and control over privacy bounds.

Processes required for privacy and security based on stakeholder needs and political regulations may operate as roadblocks to HIT adoption. Many safeguards (eg, safe login) must be taken by organizations, and such rules considerably reduce the availability of privacy-sensitive information on the portal, affecting data quality [61].

#### Individual (HCP)-Readiness to Implementation (Inner Setting)

There were various individual factors of HCPs that challenged the successful implementation of HIT. HCPs indicated that they have a lack of time [48,49,51,53,58,61,66,73], they did not have enough time to adjust [48,49,53,58], or the use of HIT increased consultation time and therefore depleted time resources [51,73]. HCPs often had competing priorities [58,79] in work and perceived the newly implemented HIT as noncore work activity [58].

The most frequently mentioned facilitator was training [40,50,73], and succinct and customized information was valued [50].

#### Individual (HCP)-Characteristics of Individuals

Individual characteristics that held up implementation were lack of knowledge [51], past negative experience [50,63], resistance toward change [50], and poor communication style [47]. Sometimes physicians preferred traditional health care messages [79] and thought that change is unneeded [51], especially because they did not believe patients could not efficiently manage their data [66].

In contrast, having a good communication style (eg, friendly and sympathetic) [47] and a good attitude toward HIT implementation acted as a facilitator [61].

#### Interpersonal

Many facilitators acted through interpersonal relationships. Prior experience from other HCPs provided legitimacy and had a positive influence via professional and social networks [53,59].

For patients, physician guidance [47,48,75], recommendations [85], and feedback from HCPs [80] assisted them in using HIT and made them feel supported [53]. Patients were more likely to use HIT when it was recommended by trusted physicians [72]. Patients benefited from family support as well [74]. It operated as a barrier when the need for long-term guidance by HCPs or family members was not adequately addressed [41,43,49]. The introduction of HIT was also hampered by a lack of connection with peers (patients) [41] and a lack of trust in communicating with HCPs [56,90].

In addition, cooperation between HCPs and various stakeholders was important. HCPs stated that a team approach to decision-making [53] and sharing information between providers was useful [44].

However, a lack of coordination between vendors and the hospital [57], nurses, and providers challenged the implementation process [58]. Since interpersonal factors play an important role in HIT implementation, it was recommended to leverage existing relationships to gain momentum [59].

#### Organizational-Inner Setting

Underlying organizational issues [55,73,78,87] and unclear responsibility of HCPs [58,65,90] created confusion. Lack of fit with existing workflow was frequently stated [51,55,79]. When new technology did not match existing practice routines or clinic schedules, the start-up period of HIT implementation was associated with an initial drop in productivity [55]. In contrast, HIT implementation matching the workflow acted as a facilitator [58,64,76]. This highlights the importance of incorporating an optimal workflow strategy [79].

The readiness of an organization to implement HIT also played a significant role. For example, lack of administrative support [77], lack of infrastructure and equipment [40,48,57,64,87,88], lack of financial resources [48,61,87], and lack of workforce [48,61,87] were noted as barriers. Conversely, administrative support [55,61], adequate infrastructure [57,58] (eg, computer resources), adequate financial resources [50], and technical support [55] were facilitators.

#### Political-Outer Setting

External policies at the political level had an impact as well. Stakeholders stated their concerns with the Health Insurance Portability and Accountability Act (HIPAA) regulations [52], which govern the privacy and security of personal data. There may also be some delays in the implementation of HIT that may benefit organizations owing to government policies [87]. Facilitating rules and regulations can be advantageous, as evidenced by the support for portal implementation by the Netherlands government [61]. On the other hand, deploying HIT was hampered by a lack of government and health care system support [67].

## Discussion

### Principal Findings

This review identified various barriers and facilitators of the implementation of HIT programs for NCD management. We conducted the analysis in 2 parts. In part 1, we focused on the inherent characteristics of HIT interventions. A relative advantage to the existing health care system was most frequently reported as a facilitator. Especially, convenience, improvement of the quality of care, and improvement in accessibility were considered useful. Design quality and usability issues, such as difficulty in using the system and data quality, were the most prominent barriers. Tackling these practical issues would be crucial in the implementation process.

In part 2, we used the novel integrated framework to indicate the human factors of implementation. Individual factors of patients related to self-efficacy were the most noted barriers. Adequate knowledge of the health system, medical data, and adequate health literacy acted as facilitators. HCPs often indicated that they have a lack of time, while training was the most quoted facilitator. At the interpersonal level, the social relationships that support the implementation process were crucial, such as the prior experience of peers, communication with HCPs, and support from family members. At the organizational level, lack of fit with existing workflow acted as a barrier, while adequate infrastructure, technical support, and financial resources were facilitators. At the political level, regulation concerns were mentioned, but facilitating rules and regulations can help implementation.

Therefore, internal technology factors of HIT and external human factors of stakeholders are both very important to the implementation. Policymakers and relevant stakeholders should not focus on only 1 side but recognize all aspects of change to maximize the probability of success.

### Comparison With Prior Work

Our findings concur with other reviews on the implementation of HIT [20,22,91,92]. Yet, previous reviews did not focus on NCD management and mostly listed the barriers and facilitators without structurization. For example, Finkelstein et al [92] mentioned 9 barriers (lack of usability, old age, education, cognitive impairment, workflow issues, etc) and 9 facilitators (perceived usefulness, efficiency, availability, etc) of HIT for patient-centered care. The importance of health literacy and being able to use the software has also been mentioned [10,93]. The interpersonal, organizational, and political factors we identified are in line with other studies that emphasized the importance of social relationships and human factors. For instance, a review on digital health interventions stated that social support affects patient engagement and recruitment [94]. However, 1 study reported that social influences have no significant effects on health care technology acceptance [95]. Further studies should try to understand the extent and pathway of social relationships in HIT implementation.

Usability has been emphasized as a critical factor in other HIT-related studies. A recent analysis financed by the Agency for Healthcare Research and Quality found significant flaws in the procedures, methods, and application of standards and best practices in the areas of usability and human aspects among certified EHR vendors [96]. EHRs must be used efficiently and effectively as they increasingly become a major tool for patient care. Moreover, usability difficulties for HIT in NCD management are consistent with existing usability research. One of the most used usability evaluation tools in information technology is the Health Information Technology Usability Evaluation Scale (Health-ITUES) [97]. Although the original Health-ITUES focused on mHealth technology, several aspects of our analysis overlap. “Improving the quality of life,” “having positive influence,” and “perceived usefulness” were mentioned as relative advantages for HIT in our study. Concepts related to the category “perceived ease of use” and “user control” were coded to the CFIR construct “design quality and usability.” This resemblance emphasizes the importance of usability difficulties in the acceptance of new technologies.

The individual barriers identified in this review are consistent with the analysis of Sun et al regarding what can aggravate the digital divide (limited technical infrastructure, lack of digital literacy, financial resources, and lack of access to digital hardware) [98]. The UN Secretary-General’s high-level panel on digital cooperation has also warned of rapid digitization leaving marginalized people behind [99]. The shortage of digital infrastructure in developing countries makes it vital to put the digital divide in context when developing HIT-related health policies, considering that only 45% of people are connected to the internet in developing countries [100]. The age-related digital divide is also an emerging problem. As our review and other reports have shown [43,46,86,92], many older patients fear technology and need detailed guidance. Policymakers should not neglect these issues of inequality and should pay attention to the underlying socioeconomic conditions in every step of the planning and implementation of HIT.

We have included the construct “privacy and confidentiality” within the “inner setting” of our integrated framework. The issue of privacy is a heated discussion in studies on information technology. The problem of dealing with personal health information has been identified in many countries, and the current legal framework is sometimes hard to match with the system [101]. This has affected the new legislation, for example, the HIPAA in the United States in 2013 and the General Data Protection Act in the European Union in 2016. Organizations could discuss deidentification methods of health information such as anonymization and pseudonymization. The acquisition of consent is also a complicated issue. For example, the usage of data should be differentiated depending on whether patients agreed to give their medical information for only treatment or for both research and treatment purposes. For now, “opt-in” (users taking affirmative action to offer their consent) is standard. “Opt-out” choices from national data (users taking action to withdraw consent) have also been offered in the United Kingdom [102], and this could also be considered in future HIT implementations.

Five research gaps have been identified through this review. First, most studies only mentioned patients and physicians. Other stakeholders, such as vendors, service providers, government officials, and administrative workforces, should be addressed in future research. Second, a great majority of HIT interventions targeted the diabetes population. This may be expected since diabetes involves the strictest self-management, such as weekly blood glucose testing. Nonetheless, there is an evident lack of research on the management of other chronic diseases, such as obesity and mental diseases. Further research in this area is warranted. Third, little evidence exists on the challenges of the long-term implementation of programs. Most studies included in this review covered implementations that were followed up for a short term. Fourth, the included studies might have been biased in the selection of study participants because they rarely used random sampling. More rigorous methods should be used, and response rates and reasons for unavailability or decline of participation should be reported. In addition, as our prior discussion on the digital divide implies, participants who have access to ongoing HIT programs might be inclined to have a higher socioeconomic status. Therefore, further studies should consider how to sufficiently represent older, socioeconomically disadvantaged, and other underrepresented groups. The final gap results from the underrepresentation of various countries, which may limit the generalizability of our findings. Most studies were conducted in the United States and other high-income countries. Extensive research on the implementation strategies of HIT in LMICs is necessary.

### Strengths and Limitations

This review has several strengths. First, to the best of our knowledge, this review is the first systematic review on the topic of HIT for NCD management. Second, our search strategy included as many eligible studies as possible, and double screening was performed at all stages. Third, we developed the integrated framework based on 2 widely recognized frameworks [26,37], which are comprehensive and detailed. Fourth, the quality of studies was assessed, but we did not restrict the inclusion of studies based on quality in order to capture as much literature as possible.

There were some limitations of this study. First, although the quality of the included studies was generally good, some studies were of low quality. The low-quality studies were not used to draw conclusions and had little effect on our overall findings. Second, since the included studies were about different types of HIT interventions and stakeholders, there could be limitations in applying the results to a specific setting. Finally, the perceived importance of facilitators and barriers in this study may not always correspond with the actual importance, and some factors may be more hypothetical. The reported factors may also have been influenced by publication bias.

### Conclusions

Internal factors of HIT and external human factors of implementation interplay in the implementation of HIT for chronic disease management. Among the characteristics of the intervention, having a relative advantage over existing health care was the most noted facilitator, while poor usability was the most reported barrier. In our stakeholder analysis undertaken by the integrated framework, health literacy and lack of digital skills were identified as key barriers. Various interpersonal and organizational factors were crucial (eg, physicians’ suggestions, cooperation, adequate management of data, and addressing privacy concerns). Implementation strategies of HIT could be improved by studying these barriers and facilitators. Further research should focus on studying various stakeholders, such as service providers and administrative workforces; various disease populations, such as those with obesity and mental diseases; and various countries, including LMICs.

## Appendix

Multimedia Appendix 1 PRISMA checklist.

Multimedia Appendix 2 Search syntax.

Multimedia Appendix 3 Data extraction form.

Multimedia Appendix 4 Quality appraisal domains by study methodology.

Multimedia Appendix 5 Critical appraisal of studies.

Multimedia Appendix 6 Enhancing Transparency in Reporting the Synthesis of Qualitative Research checklist.

Multimedia Appendix 7 Study characteristics.

Multimedia Appendix 8 Descriptive theme definitions and representative quotes.

Multimedia Appendix 9 Barriers and facilitators of the implementation of health information technology by the integrated framework.

## Abbreviations

CFIR: Consolidated Framework for Implementation Research
EHR: electronic health record
HCP: health care provider
Health-ITUES: Health Information Technology Usability Evaluation Scale
HIPAA: Health Insurance Portability and Accountability Act
HIT: health information technology
LMIC: low- and middle-income country
NCD: noncommunicable disease
WHO: World Health Organization
