# Supplementary material for: Using an Integrated Framework to Investigate the Facilitators and Barriers of Health Information Technology Implementation in Noncommunicable Disease Management: Systematic Review
Source: J Med Internet Res. 2022 Jul 20;24(7):e37338. doi: 10.2196/37338 (PMC9350822; doi:10.2196/37338)
Supplement: Multimedia Appendix 1 [file jmir_v24i7e37338_app1.docx]

**Multimedia Appendix 1. PRISMA Checklist** [1]

| Section/topic | # | Checklist item | Reported on page # |
| --- | --- | --- | --- |
| **Title** | | | |
| Title | 1 | Identify the report as a systematic review, meta-analysis, or both. | 1 |
| **Abstract** | | | |
| structured summary | 2 | Provide a structured summary including, as applicable: background; objectives; data sources; study eligibility criteria, participants, and interventions; study appraisal and synthesis methods; results; limitations; conclusions and implications of key findings; systematic review registration number. | 1 |
| **Introduction** | | | |
| Rationale | 3 | Describe the rationale for the review in the context of what is already known. | 2 |
| Objectives | 4 | Provide an explicit statement of questions being addressed with reference to participants, interventions, comparisons, outcomes, and study design (PICOS). | 3 |
| **Methods** | | | |
| Protocol and registration | 5 | Indicate if a review protocol exists, if and where it can be accessed (e.g., Web address), and, if available, provide registration information including registration number. | 4 |
| Eligibility criteria | 6 | Specify study characteristics (e.g., PICOS, length of follow-up) and report characteristics (e.g., years considered, language, publication status) used as criteria for eligibility, giving rationale. | 4, Multimedia Appendix 3 |
| Information sources | 7 | Describe all information sources (e.g., databases with dates of coverage, contact with study authors to identify additional studies) in the search and date last searched. | 4 |
| Search | 8 | Present full electronic search strategy for at least one database, including any limits used, such that it could be repeated. | Multimedia Appendix 2 |
| Study selection | 9 | State the process for selecting studies (i.e., screening, eligibility, included in systematic review, and, if applicable, included in the meta-analysis). | 4, Multimedia Appendix 3, Figure 3 |
| Data collection process | 10 | Describe method of data extraction from reports (e.g., piloted forms, independently, in duplicate) and any processes for obtaining and confirming data from investigators. | 4, 5 |
| Data items | 11 | List and define all variables for which data were sought (e.g., PICOS, funding sources) and any assumptions and simplifications made. | 4, Multimedia Appendix 3 |
| Risk of bias in individual studies | 12 | Describe methods used for assessing risk of bias of individual studies (including specification of whether this was done at the study or outcome level), and how this information is to be used in any data synthesis | 4, 5, Multimedia Appendix 4, Appendix 5 |
| Summary measures | 13 | State the principal summary measures (e.g., risk ratio, difference in means). | 6, Multimedia Appendix 7 |
| Synthesis of results | 14 | Describe the methods of handling data and combining results of studies, if done, including measures of 3 consistency (e.g., I2) for each meta-analysis. | 5, 6 |

1. Moher, D., et al., Preferred Reporting Items for Systematic Reviews and Meta-Analyses: The PRISMA Statement. PLOS Medicine, 2009. 6(7): p. e1000097.
