# Supplementary material for: Using an Integrated Framework to Investigate the Facilitators and Barriers of Health Information Technology Implementation in Noncommunicable Disease Management: Systematic Review
Source: J Med Internet Res. 2022 Jul 20;24(7):e37338. doi: 10.2196/37338 (PMC9350822; doi:10.2196/37338)
Supplement: Multimedia Appendix 2 [file jmir_v24i7e37338_app2.docx]

**Multimedia Appendix 2. Search syntax**

| **MEDLINE** | | | | |
| --- | --- | --- | --- | --- |
| **No** | **PICO** |  | **Query** | **Items found** |
| **1** | **Population** | Noncommunicable diseases (NCD) | "noncommunicable diseases"^3^ OR "NCD"[tiab] OR "noncommunicable diseases"[tiab] OR “chronic diseases”[tiab] OR "cardiovascular diseases"[tiab] OR cancers*[tiab] OR "chronic respiratory diseases"[tiab] OR diabetes*[tiab] OR “chronic kidney diseases”[tiab] OR “coronary artery diseases”[tiab] OR hypertension[tiab] OR hyperlipidemia[tiab] | 1,368,731 |
| **2** | **Intervention** | Health Information Technology (HIT) | Health Information Technology OR Health Information Technology[tiab] OR Electronic Health Records [tiab] OR personal health records[tiab] OR electronic prescribing [tiab] | 616,249 |
| **3** | **Outcome** | Barriers  Facilitators | Facilitator*[tiab] OR barrier* [tiab] OR challenge*[tiab] OR success[tiab] OR failure[tiab] | 2,146,230 |
| **4** |  | Stakeholder | stakeholder[tiab] OR stakeholders[tiab] OR actor[tiab] OR actors[tiab] | 72,968 |
| **6** | **Exclusion criteria** | Research type | (editorial OR news OR case reports) | 3,079,306 |
| **7** |  |  | case report[ti] | 278,650 |
| **8** | **Search Result** | #1 AND #2 AND (#3 OR #4) NOT (#6 OR #7) | | 5107 |

**Cochrane**

| **No** | **PICO** |  | **Query** | **Items found** |
| --- | --- | --- | --- | --- |
| **1** | **Population** | Noncommunicable diseases (NCD) | MeSH descriptor: noncommunicable diseases OR chronic diseases OR NCD OR cardiovascular diseases OR cancers OR chronic respiratory diseases OR diabetes OR chronic kidney diseases OR coronary artery diseases OR hypertension OR hyperlipidemia | 205,911 |
| **2** | **Intervention** | Health Information Technology (HIT) | MeSH descriptor: Health Information Technology OR Electronic Health Records OR personal health records OR electronic prescribing | 20,949 |
| **3** | **Outcome** | Barriers  Facilitators | (Facilitator* OR barrier* OR challenge* OR success OR failure) ti,ab,kw | 185,149 |
| **4** |  | Stakeholder | (stakeholder OR stakeholders OR actor OR actors) ti,ab,kw | 3,448 |
| **6** | **Exclusion criteria** | Research type | (editorial OR editorials OR news OR case reports) pt | 4,911 |
| **7** |  |  | case report[ti] | 308 |
| **8** | **Search Result** |  | #1 AND #2 AND (#3 OR #4) NOT (#6 OR #7) | 1,487 |

**EMBASE**

| **No** | **PICO** |  | **Query** | **Items found** |
| --- | --- | --- | --- | --- |
| **1** | **Population** | Noncommunicable diseases (NCD) | noncommunicable diseases OR NCD OR cardiovascular diseases OR chronic diseases OR cancers OR chronic respiratory diseases OR diabetes OR chronic kidney diseases OR coronary artery diseases OR hypertension OR hyperlipidemia: ti,ab,kw |  |
| **2** | **Intervention** | Health Information Technology (HIT) | ‘Health Information Technology’ OR ‘Electronic Health Records’ OR ‘personal health records’ OR ‘electronic prescribing’: ti, ab, kw |  |
| **3** | **Outcome** | Barriers  Facilitators | Facilitator OR ‘barrier’ OR ‘challenge’ OR ‘success’ OR ‘failure’:ti,ab,kw |  |
| **4** |  | Stakeholder | ‘stakeholder’ OR ‘stakeholders’ OR ‘actor’ OR ‘actors’:ti,ab,kw |  |
| **6** | **Exclusion criteria** | Research type | ‘editorials’ OR ‘news’ OR ‘case reports’:it |  |
| **7** |  |  | case report[ti] |  |
| **8** | **Search Result** | ('noncommunicable diseases' OR NCD OR 'cardiovascular diseases' OR 'chronic diseases' OR cancers OR 'chronic respiratory diseases' OR diabetes OR 'chronic kidney diseases' OR 'coronary artery diseases' OR hypertension OR hyperlipidemia):ti,ab,kw AND (('Health Information Technology' OR 'Electronic Health Records' OR 'personal health records' OR 'electronic prescribing'):ti,ab,kw) AND ((facilitator OR barrier OR challenge OR success OR failure OR stakeholder OR stakeholders OR actor OR actors):ti,ab,kw) NOT ((editorials OR news OR 'case reports'):it) NOT (('case report'):ti) | | 570 |

**Scopus**

| **No** | **PICO** |  | **Query** | **Items found** | | |
| --- | --- | --- | --- | --- | --- | --- |
| **1** | **Population** | Noncommunicable diseases (NCD) | “noncommunicable disease*” OR “NCD” OR “cardiovascular disease*” OR “chronic diseases*” OR “cancer*” OR “chronic respiratory disease*” OR “diabetes*” OR “chronic kidney disease*” OR “coronary artery disease*” OR “hypertension*” OR “hyperlipidemia*”: ti,ab,kw | | |  |
| **2** | **Intervention** | Health Information Technology (HIT) | “Health Information Technology*” OR “Electronic Health Records*” OR “personal health records*” OR “electronic prescribing*”: ti, ab, kw | |  | |
| **3** | **Outcome** | Barriers  Facilitators | “Facilitator*” OR “barrier*” OR “challenge*” OR “success*” OR “failure*” :ti,ab,kw | |  | |
| **4** |  | Stakeholder | “stakeholder*” OR “actor*” :ti,ab,kw | |  | |
| **5** | **Exclusion criteria** | Research type | Conference paper, Editorial, Note, Letter, Book Chapter, Conference Review, Short Survey, Book | |  | |
| **6** | **Search Result** | ( TITLE-ABS-KEY ( "Health Information Technology*" OR "Electronic Health Records*" OR "personal health records*" OR "electronic prescribing*" ) AND TITLE-ABS-KEY ( "noncommunicable disease*" OR "NCD" OR "cardiovascular disease*" OR "chronic diseases*" OR "cancer*" OR "chronic respiratory disease*" OR "diabetes*" OR "chronic kidney disease*" OR "coronary artery disease*" OR "hypertension*" OR "hyperlipidemia*" : ) AND TITLE-ABS-KEY ( "Facilitator*" OR "barrier*" OR "challenge*" OR "success" OR "failure" OR "stakeholder*" OR "actor*" ) ) AND ( LIMIT-TO ( DOCTYPE , "ar" ) OR LIMIT-TO ( DOCTYPE , "re" ) ) | | 1804 | | |

**CINAHL**

| **No** | **PICO** |  | **Query** | **Items found** |
| --- | --- | --- | --- | --- |
| **1** | **Population** | Noncommunicable diseases (NCD) | “noncommunicable disease*” OR “NCD*” OR “cardiovascular disease*” OR “chronic diseases*” OR “cancer*” OR “chronic respiratory disease*” OR “diabetes*” OR “chronic kidney disease*” OR “coronary artery disease*” OR “hypertension*” OR “hyperlipidemia*” | |
| **2** | **Intervention** | Health Information Technology (HIT) | “Health Information Technology*” OR “Electronic Health Records*” OR “personal health records*” OR “electronic prescribing*” | |
| **3** | **Outcome** | Barriers  Facilitators | “Facilitator*” OR “barrier*” OR “challenge*” OR “success*” OR “failure*” | |
| **4** |  | Stakeholder | “stakeholder*” OR “actor*” | |
| **5** | **Publication** | Research type | Peer Reviewed Academic Journals (Exclude magazines) |  |
| **6** | **Search Result** | Boolean: #1 AND #2 AND (#3 OR #4), Limiters: Peer Reviewed Academic Journals | | 2734 |
