# Supplementary material for: Using an Integrated Framework to Investigate the Facilitators and Barriers of Health Information Technology Implementation in Noncommunicable Disease Management: Systematic Review
Source: J Med Internet Res. 2022 Jul 20;24(7):e37338. doi: 10.2196/37338 (PMC9350822; doi:10.2196/37338)
Supplement: Multimedia Appendix 3 [file jmir_v24i7e37338_app3.docx]

Multimedia Appendix 3. Data collection form

# Study eligibility

| Study Characteristics | 1. Inclusion criteria 2. Exclusion criteria | | Eligibility criteria met? | | | Location in text or source |
| --- | --- | --- | --- | --- | --- | --- |
|  |  |  | Yes | No | Unclear |  |
| Participants | 1. Stakeholders related to NCD management  2. Studies on HIT that does not focus on NCD management | |  |  |  |  |
| Types of intervention | 1. Any health intervention delivered by health information technology  This includes, but is not limited to: Web-based interventions, Patient portals, personal health records, electronic health records, and electronic prescribing  2. Studies that focus on telehealth or mHealth (e.g. mobile application with scarce HIT function) | |  |  |  |  |
| Types of outcome measures | 1. Studies that examine implementation outcomes (barriers and facilitators) 2. Studies that do not discuss the relevant outcome measures (e.g. studies exploring the effectiveness of HIT, opinions or perspectives with scarce evidence) | |  |  |  |  |
| Methods | 1. Original studies with direct contact or direct observation of stakeholders  2. Secondary studies and reviews | |  |  |  |  |
| Publication and language | 1. English, published in peer-reviewed academic journals  2. Published abstracts, conference proceedings, or descriptive case studies | |  |  |  |  |
| INCLUDE | | EXCLUDE | | | | |
| Reason for exclusion |  | | | | | |

**DO NOT PROCEED IF STUDY EXCLUDED FROM REVIEW**

# Characteristics of included studies

## Methods

|  | **Descriptions as stated in report/paper** | | **Location in text or source** |
| --- | --- | --- | --- |
| **Author, year, country** | *Name of authors. e.g. Janssen et al.* | |  |
| **Design** | *qualitative, quantitative, mixed-method, survey etc.* | |  |
| **Data collection methods** |  | |  |
| **Target population** |  | |  |
| **Stage of intervention** |  | |  |
| **Health Information Technology Intervention** |  | |  |
| **Addressed stakeholders** |  | |  |
| **Ethical approval needed/ obtained for study** | Yes No Unclear |  |  |

## Participants

|  | **Description** | **Location in text or source** |
| --- | --- | --- |
| **Population description** *(from which study participants are drawn)* |  |  |
| **Setting** *(including location and social context)* |  |  |

## Other

| **Possible conflicts of interest** *(for study authors)* |  |  |
| --- | --- | --- |

# Other information

|  | **Description as stated in report/paper** | **Location in text or source** |
| --- | --- | --- |
| **Key conclusions of study authors** |  |  |
| **References to other relevant studies** |  |  |
