# Supplementary material for: Using an Integrated Framework to Investigate the Facilitators and Barriers of Health Information Technology Implementation in Noncommunicable Disease Management: Systematic Review
Source: J Med Internet Res. 2022 Jul 20;24(7):e37338. doi: 10.2196/37338 (PMC9350822; doi:10.2196/37338)
Supplement: Multimedia Appendix 4 [file jmir_v24i7e37338_app4.docx]

**Multimedia Appendix 4. Quality Appraisal Domains by Study Methodology**

| **Qualitative – Joanna and Briggs Tool**  **Domain 1: Design and methodology**  Q1. Is there congruity between the stated philosophical perspective and the research methodology?  Q2. Is there congruity between the research methodology and the research question or objectives?  Q3. Is there congruity between the research methodology and the methods used to collect data?  Q4. Is there congruity between the research methodology and the representation and analysis of data?  Q5. Is there congruity between the research methodology and the interpretation of results?  **Domain 2: Researcher influence**  Q6. Is there a statement locating the researcher culturally or theoretically?  Q7. Is the influence of the researcher on the research, and vice- versa, addressed?  **Domain 3: Participants**  Q8. Are participants, and their voices, adequately represented?  Q9. Is the research ethical according to current criteria or, for recent studies, is there evidence of ethical approval by an appropriate body?  **Domain 4: Interpretation of results**  Q10. Do the conclusions drawn in the research report flow from the analysis, or interpretation, of the data?  **Mixed Methods – Mixed Methods Appraisal Tool (MMAT), version 2018**  Q1. Is there an adequate rationale for using a mixed methods design to address the research question? Q2. Are the different components of the study effectively integrated to answer the research question? Q3. Are the outputs of the integration of qualitative and quantitative components adequately interpreted? Q4. Are divergences and inconsistencies between quantitative and qualitative results adequately addressed? Q5. Do the different components of the study adhere to the quality criteria of each tradition of the methods involved?  **Survey – Center for Evidence-Based Management (CEBM)**  Q1. Did the study address a clearly focused question / issue?  Q2. Is the research method (study design) appropriate for answering the research question?  Q3. Is the method of selection of the subjects (employees, teams, divisions, organizations) clearly described?  Q4. Could the way the sample was obtained introduce (selection)bias?  Q5. Was the sample of subjects representative with regard to the population to which the findings will be referred?  Q6. Was the sample size based on pre-study considerations of statistical power?  Q7. Was a satisfactory response rate achieved?  Q8. Are the measurements (questionnaires) likely to be valid and reliable?  Q9. Was the statistical significance assessed?  Q10. Are confidence intervals given for the main results?  Q11. Could there be confounding factors that haven’t been accounted for?  Q12. Can the results be applied to your organization? |
| --- |
