# Supplementary material for: Using an Integrated Framework to Investigate the Facilitators and Barriers of Health Information Technology Implementation in Noncommunicable Disease Management: Systematic Review
Source: J Med Internet Res. 2022 Jul 20;24(7):e37338. doi: 10.2196/37338 (PMC9350822; doi:10.2196/37338)
Supplement: Multimedia Appendix 5 [file jmir_v24i7e37338_app5.docx]

**Multimedia Appendix 5. Critical Appraisal of Studies**

**Table S1. Critical Appraisal of Qualitative Studies**

|  | **Domain 1: Design and Methodology** | | | | | **Domain 2: Researcher Influence** | | **Domain 3: Participants** | | **Domain 4: interpretation of results** | **Overall Quality** | |
| --- | --- | --- | --- | --- | --- | --- | --- | --- | --- | --- | --- | --- |
|  | **Q1** | **Q2** | **Q3** | **Q4** | **Q5** | **Q6** | **Q7** | **Q8** | **Q9** | **Q10** | |  |
| Abidi et al., (2018) | Yes | Yes | Yes | Yes | Yes | Yes | Yes | Yes | Yes | Yes | | High |
| Ancker et al. (2015) | Yes | Yes | Yes | Yes | Yes | Yes | No | Yes | Yes | Yes | | High |
| Ancker et al. (2015) | Yes | Yes | Yes | Yes | Yes | Yes | No | Yes | Yes | Yes | | High |
| Baudendistel, et al. (2015) | Yes | Yes | Yes | Yes | Yes | Yes | No | Yes | Yes | Yes | | High |
| Dehnavi et al. (2021) | Yes | Yes | Yes | Yes | Yes | Yes | Yes | Yes | Yes | Yes | | High |
| Desai et al. (2022) | Yes | Yes | Yes | Yes | Yes | Yes | Yes | Yes | No | Yes | | High |
| Dikomitis et al. (2015) | Yes | Yes | Yes | Yes | Yes | Yes | No | Yes | Yes | Yes | | High |
| Fontil et al. (2016) | Yes | Yes | Yes | Yes | Yes | Yes | No | Yes | Yes | Yes | | High |
| Fuji et al. (2015) | Yes | Yes | Yes | Yes | Yes | No | No | Yes | No | Yes | | Medium |
| Groenhof et al. (2019) | Yes | Yes | Yes | Yes | Yes | Yes | No | not clear | not clear | Yes | | High |
| Hans et al. (2018) | Yes | Yes | Yes | Yes | Yes | Yes | Yes | Yes | Yes | Yes | | High |
| Heider et al. (2014) | not clear | not clear | not clear | not clear | Yes | Yes | not applicable | not clear | No | Yes | | Low |
| Hess et al. (2007) | Yes | Yes | Yes | Yes | Yes | Yes | Yes | Yes | No | not clear | | High |
| Janssen et al. (2021) | Yes | Yes | Yes | Yes | Yes | Yes | No | Yes | Yes | Yes | | High |
| Kooij et al., (2018) | Yes | Yes | Yes | Yes | Yes | Yes | Yes | Yes | No | Yes | | High |
| Lober at al. (2006) | Yes | Yes | Yes | Yes | Yes | Yes | No | No | Yes | Yes | | High |
| Marchak et al. (2019) | Yes | Yes | Yes | Yes | Yes | No | No | Yes | Yes | Yes | | High |
| Marquard et al. (2013) | Yes | Yes | Yes | Yes | Yes | Yes | No | No | No | Yes | | Medium |
| McBride, et al. (2014) | Yes | Yes | Yes | Yes | Yes | No | No | No | No | Yes | | Medium |
| Portz et al, (2019) | Yes | Yes | Yes | Yes | Yes | Yes | No | Yes | No | Yes | | High |
| Pratt et al, (2021) | Yes | Yes | Yes | Yes | Yes | Yes | Yes | Yes | Yes | Yes | | High |
| Ralston et al. (2004) | yes | Yes | Yes | Yes | Yes | Yes | Yes | Yes | Yes | Yes | | High |
| Saleem et al. (2005) | Yes | Yes | Yes | Yes | Yes | No | No | Yes | No | Yes | | Medium |
| Solberg et al. (2017) | not clear | not clear | not clear | Yes | Yes | No | No | No | No | Yes | | Low |
| Tieu et al. (2015) | Yes | Yes | Yes | Yes | Yes | Yes | No | Yes | No | Yes | | High |
| Tong et al. (2020) | Yes | Yes | Yes | Yes | Yes | Yes | No | Yes | Yes | Yes | | High |
| Tong et al. (2020) | Yes | Yes | Yes | Yes | Yes | Yes | Yes | Yes | Yes | Yes | | High |
| Trivedi et al. (2009) | Yes | Yes | Yes | Yes | Yes | Yes | No | No | No | Yes | | Medium |
| Urowitz et al. (2012) | Yes | Yes | Yes | Yes | Yes | No | No | Yes | Yes | Yes | | High |
| Varonen et al. (2008) | Yes | Yes | Yes | Yes | Yes | No | Yes | Yes | Yes | Yes | | High |
| Wan et al. (2012) | Yes | Yes | Yes | Yes | Yes | No | No | Yes | Yes | Yes | | High |
| Wang et al. (2013) | Yes | Yes | Yes | Yes | Yes | Yes | No | Yes | Yes | Yes | | High |
| Yu et al. (2019) | Yes | Yes | Yes | Yes | Yes | No | No | Yes | Yes | Yes | | High |
| Zwaanswijk et al. (2013) | Yes | Yes | Yes | Yes | Yes | Yes | Yes | Yes | No | Yes | | High |

**Domain 1: Design and methodology -** High quality (green) = 4 or more yeses; Medium quality (orange) = 3 yeses; Low quality (red) = 2

**Domain 2: Researcher influence -** High quality (green) = 2 yeses; Medium quality (orange) = 1 yes; Low quality (red) = 0 yeses.

**Domain 3: Participants -** High quality (green) = 2 yeses; Medium quality (orange) = 1 yes; Low quality (red) = 0 yeses.

**Domain 4: Interpretation of results -** High quality (green) = 1 yes; Low quality (red) = 0 yeses.

**Overall Quality**- High quality (Green) = 8~10 yeses; Medium quality (orange) = 4~7 yeses; Low quality(red) = 1~3 yeses

**Table S2. Critical Appraisal of Mixed Methods Studies**

|  | Q1 | Q2 | Q3 | Q4 | Q5 | Quality |
| --- | --- | --- | --- | --- | --- | --- |
| Brown et al. (2017) | Yes | Yes | Yes | Yes | No | Medium |
| Conway et al. (2019) | No | Yes | Yes | Yes | Yes | Medium |
| Diaz-Garelli et al. (2021) | No | Yes | No | Yes | Yes | Medium |
| Dixon et al. (2013) | Yes | Yes | Yes | Yes | No | Medium |
| Grant et al. (2015) | No | Yes | Yes | Yes | No | Medium |
| Jethwani et al. (2012) | Yes | Yes | Yes | Yes | No | Medium |
| Marcolino et al. (2021) | Yes | Yes | Yes | Yes | Yes | High |
| Mayberry et al. (2011) | Yes | Yes | Yes | Yes | Yes | High |
| Osborn et al. (2013) | No | Yes | Yes | Yes | Yes | Medium |
| Pemu et al. (2013) | Yes | Yes | Yes | Yes | No | Medium |
| Tieu et al. (2016) | Yes | Yes | Yes | Yes | No | Medium |
| Wade-Vuturo et al. (2013) | Yes | Yes | Yes | Yes | Yes | High |
| Wildeboer et al. (2018) | Yes | Yes | Yes | Yes | Yes | High |

Yes = green; No = red; High quality (green) = 5 yeses; Medium quality (orange) = 3~4 yeses

**Table S3. Critical Appraisal of Quantitative and Survey Studies**

| **survey/quantitative** | Q1 | Q2 | Q3 | Q4 | Q5 | Q6 | Q7 | Q8 | Q9 | Q10 | Q11 | Q12 | Quality |
| --- | --- | --- | --- | --- | --- | --- | --- | --- | --- | --- | --- | --- | --- |
| Allain et al., (2017) | Yes | Yes | Yes | Yes | Yes | No | No | Yes | Yes | No | Yes | Yes | Medium |
| Kabukye et al. (2020) | Yes | Yes | Yes | Yes | Yes | No | Yes | Yes | Yes | No | Yes | Yes | Medium |
| Ronda et al. (2014) | Yes | Yes | Yes | Yes | Yes | No | No | Yes | Yes | Yes | Yes | Yes | Medium |
| Sarkar et al. (2010) | Yes | Yes | Yes | Yes | Yes | No | No | Yes | Yes | Yes | Yes | Yes | Medium |

Q1, Q2, Q3, Q5, Q6, Q7, Q8, Q9, Q10, Q12- Yes = green, No = red; Q4, Q11 – Yes = red, No = green

High quality (green) = more than 10 greens; Medium quality (orange) = 7~9 yeses
