# Supplementary material for: Using an Integrated Framework to Investigate the Facilitators and Barriers of Health Information Technology Implementation in Noncommunicable Disease Management: Systematic Review
Source: J Med Internet Res. 2022 Jul 20;24(7):e37338. doi: 10.2196/37338 (PMC9350822; doi:10.2196/37338)
Supplement: Multimedia Appendix 7 [file jmir_v24i7e37338_app7.docx]

**Multimedia Appendix 7. Study characteristics**

| 1. Author, Year, Country  2. Quality Rating | 1. Study Design 2. Data collection methods | Participants | Stage of intervention | Target populations | Health Information Technology Program or intervention | Addressed stakeholders |
| --- | --- | --- | --- | --- | --- | --- |
| 1. Abidi et al., 2018, Canada [65] 2.100% | 1. Qualitative 2. record oarticipants’ screen activity and audio, field notes, and observation logs (n=11), focus group interview (n=7) | 10 PCPs (4 family physicians and 6 CDEs) for the PCP study and 11 patients | evaluation of intervention | Diabetes (T2DM) | Computerized decision support “Diabetes Web-Centric Information and Support Environment” (DWISE), assists primary care practitioners in applying standardized behavior change strategies and clinical practice guidelines–based recommendations to an individual patient and empower the patient with the skills and knowledge required to self-manage their diabetes through planned, personalized, and pervasive behavior change strategies. | patients, providers |
| 1. Allain et al., 2017, Malawi [88] 2. 66% | 1. Quantitative 2. extracted from the Diabetes Clinic EMRs (n=1112) | patients with a confirmed diagnosis of diabetes mellitus (DM) seen at the Queen Elizabeth Central Hospital (QECH) diabetes | evaluation of intervention | diabetes(T1DM + T2DM) | touch-screen based electronic medical record system (EMRs) | patients, providers |
| 1. Ancker et al., 2015, US [38] 2. 90% | 1. Qualitative 2. semi-structured interviews (n=29) | 22 patients and 7 providers recruited from New York-Presbyterian Hospital and the Institute for Family Health | before intervention | people with multiple chronic conditions (MCC) | personal health information management (PHIM) | patients, providers |
| 1. Ancker et al., 2015, USA [39] 2. 90% | 1. Qualitative 2. semi-structured interviews (n=29) | 22 patients and 7 providers recruited from New York-Presbyterian Hospital and the Institute for Family Health | before intervention | people with multiple chronic conditions (MCC) | personal data tracking | patients, providers |
| 1. Baudendistel, et al., 2015, Germany [66] 2. 80% | 1. Qualitative 2. focus groups semi-structured interviews (n=47) | patients with colorectal cancer (n=12) and representatives from patient support groups (n=2), physicians (n=17), and other health care professionals (HCPs) (n=16). | evaluation of intervention | patients with colorectal cancer | Personal electronic health record(PEPA), a pilot project called BInformation technology for patientcentered health care(INFOPAT), funded by the German Federal Ministry of Education and Research (2012–16), | patients |
| 1. Brown et al., 2017, USA [72] 2. 80% | 1. mixed methods 2. qualitative focus group interviews (n=35), survey | 35 women diagnosed with GDM | evaluation of intervention | Gestational Diabetes Mellitus (GDM) | program outreach messages containing content tailored to EHR-derived diabetes risk factors | patients |
| 1. Conway et al., 2019, Scotland [77] 2. 80% | 1. Quantitative 2. Online questionnaire (completed n=1096) | 1096 active, registered MDMW users | service improvement project | Diabetes (T1DM + T2DM) | ePHR (electronic Personal Health Record): the MDMW ePHR provides data on key diabetes indicators, descriptive text, educational materials, and clinical results | patients |
| 1. Dehnavi et al., 2021, Iran [67]  2. 100% | 1. Qualitative 2. semi-structured interviews (n=18) | 8 patients and 10 specialists in an endocrine and metabolism research center and in a teaching hospital | evaluation of intervention | diabetes | use of health information technology in diabetes management | patients, providers |
| 1. Desai et al., 2022, USA [68]  2. 100% | 1. Qualitative 2. individual interviews (n=110) | a wide range of disciplines and professions, as well as others involved in administration of the hospital or clinics within cancer center | evaluation of intervention | patients with cancer | Patient Values Tab, which provides ready access to these key documents, along with other information elicited by members of the interdisciplinary healthcare team illuminating the patient with cancer as a person with individual values, goals, and preferences. In a single EHR location, this Patient Values Tab organizes, displays, and highlights such information as prominently as laboratory values and other objective data that typically dominate the EHR. | patients, providers |
| 1. Diaz-Garelli et al., 2021, Malawi [84]  2. 60% | 1. mixed methods 2. Surveys (n=19) and semi structured focus group interviews（n=12） | 12 neuro-oncologists and thoracic oncologists and 19 members of the thoracic and neuro-oncology departments | evaluation of intervention | oncology healthcare providers | cancer diagnosis data entry into local EHR | Health care providers |
| 1. Dikomitis et al., 2015, UK [40] 2. 90% | 1. qualitative 2. Telephone interviews (n=23) | self-selected 23 GP(general practitioner)s | evaluation of intervention | suspected lung or colorectal cancer | electronic risk assessment tools (eRATs): consists of three components; (1) in-consultation on-screen prompts; (2) an interactive risk calculator and (3) audit tables of patients with calculated positive predictive values | general practitioners (GPs) |
| 1. Dixon et al., 2013, USA [78] 2. 80% | 1. Mixed methods 2. log file data, descriptive statistics, qualitative methods (n=3) | 3 Wishard volunteer primary care physicians | evaluation of pilot study | primary care patients | CDS rules engine hosted in the cloud: triggered preventive care reminders , returned for display to clinician end users for review and display | physicians |
| 1. Fontil et al., 2016, USA [41] 2. 90% | 1. qualitative 2. Focus groups, feasibility study, semi-structured phone interviews (n=18) | 18 low-income prediabetic patients | design, adaptation, and feasibility study of program | Diabetes (low-income prediabetes patients) | Digital diabetes prevention program (DPP): The Omada Health Program®, an Internet- and mobile-phone-based educational program. Includes small group support, personalized health coaching, a weekly curriculum, and digital tracking tools. | patients |
| 1. Fuji et al., 2015, USA [56] 2. 70% | 1. qualitative 2. Interviews (n=59) after initial training on tool | 59 patients with T2DM | assess usfage of existing program | Diabetes (T2DM) | PHR(personal health records); Microsoft HealthVault ©, features included: sharing health information, receiving feedback based on entered health information, secure messaging with healthcare providers, interoperability with providers' health records, etc. | patients |
| 1. Grant et al., 2015, USA [79] 2. 60% | 1. mixed methods  2. RCT, post intervention online or paper surveys (n=20) | 44 primary care physicians (PCPs), 20 survey participants | evaluation of intervention | primary care patients prescribed for hyperlipidemia, diabetes, and/or hypertension management over a 12-month period | a novel health IT Tool to support between-visit-based laboratory monitoring for chronic disease medication prescriptions: added features were the "medication metronome”, where the system tracked the future laboratory tests ordered by an intervention physician prescribing a study medicine. | patients, physicians |
| 1.Groenhof, et al., 2019, Netherlands [69] 2. 80% | 1. qualitative 2. Interviews (n=7) | clinicians from inside and outside the expert group (n= 7) | evaluation of intervention | people with Cardiovascular diseases | real-time computerised decision support system (CDSS) embedded in the electronic health record (EHR); provides information on cardiovascular risk factors, estimated 10-year cardiovascular risk, guideline-compliant suggestions for both pharmacological and non-pharmacological treatment to optimise risk factors, and an estimate on the change in 10-year risk of cardiovascular disease if treatment goals are adhered to. | patients, providers |
| 1.Hans, et al., 2018, Canada [70] 2. 100% | 1. qualitative 2. Interviews (n=11) | interdisciplinary primary care providers (n = 6) and their complex care patients (n = 12) | evaluation of intervention | two or more chronic conditions(MCC) | Electronic Patient-Reported Outcome (ePRO) mobile application and portal system | patients, providers |
| 1. Heider et al., 2014, USA [59]  2. 30% | 1. qualitative 2. Descriptive | 98 practices (344 providers) serving over 50,000 adult diabetic patients | developing an HER registry | diabetes | EHR registry development | vendors, providers, physicians |
| 1. Hess et al., 2007, USA [42] 2. 80% | 1. Qualitative 2. Focus group interviews(n=39) | 21 preimplementation focus group participants and 18 postimplementation focus group participants | evaluation of pilot project | diabetes | PHR(personal health records), UPMC HealthTrak is based in the physician office and connects the patient, physician, and electronic medical record (EMR). Its functionalities include secure, electronic communication with the physician’s office, preventive healthcare reminders, and disease-specific tools and information | patients |
| 1. Janssen et al., 2021, Australia [71] 2. 90% | 1. qualitative 2.face to face or via phone interviews (n=12), post-launch interviews (n=8) | 12 clinical and administrative staff of a cancer centre | evaluation of intervention (pre-implementation) | cancer | Electronic Health Records, was implemented in November 2017 in a breast cancer service within Western Sydney Local Health District. | providers |
| 1. Jethwani et al., 2012, USA [80] 2. 80% | 1. mixed methods 2. Quantitative evaluation and focus group interviews(n=20) | 75 patients from practices within Partners Healthcare system in Massachusetts; | evaluation of intervention | Diabetes (T2DM) | Diabetes Connect (DC), a Web-based diabetes self-management program, a 12-month program that allows patients to upload their blood glucose readings to a database, monitor trends, and share their data with their providers. | patients |
| 1.Kabukye et al., 2020, Uganda [87] 2. 66% | 1. survey 2. Surveys (n=116) | clinical and non-clinical staff | before or at the early stage of intervention | Oncology in LMICs | off-the-shelf EHR called Clinic Master ; used for patient registration, appointments scheduling, and retrospective capture of some clinical details such as diagnosis and treatment, as well as for tracking paper files. | providers |
| 1. Kooij et al., 2018, Netherland [61] 2. 90% | 1. qualitative 2. semistructured interviews (n=21 | medical professionals (n=7), managers (n=7), and IT employees (n=7), | evaluation of intervention | University medical center, teaching hospitals, general hospitals | patient portal: “a personal digital environment, facilitated by a health care institution, for example a hospital. Patients need to login to the portal to get access to, for example, their medical file (with results), patient information and appointments. Patients can also fill in questionnaires and receive personalized advice regarding, for example, quality of life and physical activity.” | Medical professionals, managers, IT employees |
| 1. Lober et al., 2006, USA [43] 2. 80% | 1. qualitative 2. Descriptive study (n=38) | 38 residents from HA | starting use of program | low-income elderly and disabled population, residents of a publicly subsidized housing project (HA) | personal health record, Personal Health Information Management System (PHIMS) , PHIMS displays a single category of health information at a time, and uses large font sizes, and high contrast screens. | patients |
| 1. Marchak et al., 2019, USA [44] 2. 80% | 1. qualitative 2. Focus groups and structured telephone interviews (n=28) | young adult survivors of pediatric cancer (n= 3), parents (n=11),and healthcare providers (n=14) | user-centered design and evaluation | Parents of pediatric cancer survivors and young adult survivors (aged 18–21 years) | PHR(personal health records); Cancer SurvivorLink™, along with digital evidence-based education and resources | patients, parents, health care providers |
| 1. Marcolino et al., 2021, Brazil [73]  2. 100% | 1. mixed methods 2. quasi-experimental study, likert-scale questionnaire(n=96) for usability assessment, focus-group interview (n=17) | quasi-experimental study, likert-scale questionnaire, focus-group interview (96 health care professionals) | pilot testing for implementation of program | diabetes and hypertension | clinical decision support system (CDSS) for diabetes and hypertension | patients, health care providers |
| 1. Marquard et al., 2013, USA [57] 2. 70% | 1. qualitative 2. telephone calls (n=26), home visit (n=8) | 26 patients for pilot study, home visits with 8 participants | development and refinement of intervention | diabetes and hypertension management | patient-generated data into clinical EHR: the CONDUIT-HID intervention integrates patients’ electronic blood pressure measurements directly into the clinical EHR | patient, research team |
| 1. Mayberry et al., 2011, USA [74] 2. 100% | 1. mixed methods 2. focus group discussions (n=45) and survey (n=61) | 61 adults with T2DM | usage study of HIT | Diabetes (T2DM) | Patient web portals (PWPs):MyHealthAtVanderbilt (MHAV) | patients |
| 1.McBride, et al., 2014, US [62] 2. 80% | 1. qualitative 2. Interviews (n=20) | medical directors and quality improvement champions from safety-net adult primary care clinics | evaluation of intervention | primary care provider (PCP) and patients with CKD | a CKD registry in safety-net primary care. | Patients and health care providers |
| 1. Osborn et al., 2013, USA [74] 2. 80% | 1. mixed methods 2. focus groups (n=45), surve y (n=61), medical chart review | 61 adults with T2DM | evaluation of intervention | Diabetes (T2DM) | patient web portal: MyHealthAtVanderbilt (MHAV), can manage medical bills, view PHI from HER, use secure messaging, access hospital information, etc. | patients |
| 1. Pemu et al., 2019, USA [82] 2. 80% | 1. mixed methods 2. Self-administered quantitative questionnaires, semi-structured discussions using conference calls (n=264) | 264 participants recruited from community clinics | evaluation of intervention | Diabetes (T2DM) | consumer health  information technology (CHIT): e-Healthystrides© health coach facilitated intervention. 1) PHR, 2)self-directed diabetes education, 3) email contact with clinic or health coach, 4) peer support | patients |
| 1. Portz, et al., 2019, US [63] 2. 80% | 1. qualitative 2. Interviews (n=24) | 24 older patients | evaluation of intervention | older patients with MCC. | Kaiser Permanente Colorado’s established patient portal, My Health Manager. My Health Manager provides personal health information related to patient diagnosis, prescriptions, laboratory results, and vaccination records. To improve provider-patient communication, My Health Manager offers features for patients to email providers and schedule appointments. | Patients, providers |
| 1. Pratt, et al., 2021, US [64] 2. 100% | 1. qualitative 2. telephone interviews (n=22) | 11 clinicians, 6 rooming staff, and 7 nurse or clinic managers | evaluation of intervention | adults with prediabetes | Pre-D CDS (clinical decision support): exchange EHR information with the CDS webservice, an algorithmically generated best-practice alert (BPA) appeared on the EHR screen; the CDS displays given to the PCC and the patient to review immediately prior to their visit; if appropriate, during the visit PCCs and patients review the CDS information and made shared decisions | Patients, providers |
| 1. Ralston et al., 2004, USA [45] 2. 100% | 1. Qualitative 2. Semi-structured interviews (n=9) | 9 patients from general internal medicine clinic | evaluation of intervention | Diabetes (T2DM) | diabetes care module that included access to their electronic medical record, secure email, ability to upload blood glucose readings, an education site with endorsed content, and an interactive online diary for entering exercise, diet, and medication. | patients |
| 1. Ronda et al., 2014, The Netherlands [85] 2. 60% | 1. quantitative 2. Survey (n=1390) | 1390 randomly selected users of patient portal | evaluation of intervention | Diabetes (T1DM + T2DM) | Web portal: "Digitaal Logboek", patients have access to their diabetes-specific medical records, laboratory results, general diabetes information, personal examiniations and consultations, can import and upload glucose measures, etc. | patients |
| 1. Saleem et al., 2005, USA [58] 2. 70% | 1. qualitative 2. Observational study (n=90) | 35 nurses, 55 physicians and mid-level practitioners | evaluation of intervention | general | Computerized Patient Record System (CPRS) in The Veterans Administration Computerized Patient Record System and Clinical Reminder System. The CPRS is an integrated program with multiple software packages designed to allow providers to order medications, laboratory tests, consultations, and document actions. | health care providers |
| 1. Sarkar et al., 2010, USA [86] 2. 60% | 1. quantitative 2. survey self-report items (n=14,102) | 14,102 participants from the Diabetes Study of Northern California (DISTANCE) study | evaluation of intervention | diabetes | Internet-based patient portals : KP. Org, Key features include laboratory test results with interpretation and email communication with physicians, clinical transactions of refilling medications, making medical appointments | patients |
| 1. Solberg et al., 2017, USA [60] 2. 30% | 1. qualitative 2. Case study | NA | implementation stage | depression and diabetes or heart disease | collaborative care management mode, COMPASS (Care of Mental, Physical, and Substance-use Syndromes) | care managers, medical care groups |
| 1. Tieu et al., 2016, USA [83] 2. 80% | 1. mixed methods 2. surveys , interviews and think-aloud methods (n=25) | 23 patients and 2 caregivers | performance testing of intervention | patients with chronic disease | patient portals: a Safety Net Health Care System | patients and caregivers |
| 1. Tieu et al., 2015, USA [46] 2. 80% | 1. qualitative 2. in-depth interviews (n=16) | 11 patients and 5 caregivers | pre-implementation | patients with chronic disease | pateint portal: a Safety Net Health Care System | patients and caregivers |
| 1. Tong et al., 2020, Malaysia [47] 2. 90% | 1. qualitative 2. in-depth interviews and focus group discussions (n=16) | 16 patients with T2DM who have been advised to start insulin or were currently using insulin and those who had been attending the primary care clinic regularly | implementation stage | Diabetes (T2DM) | patient decision aids (PDAs): insulin PDA that helps patients make an informed decision whether or not to iitiate insulin | patients |
| 1. Tong et al., 2020, Malaysia [48] 2. 100% | 1. qualitative 2. focus group semi-structured interview (n=43) | 28 health care providers and 15 patients | implementation stage | diabetes | patient decision aids (PDAs): insulin PDA that helps patients make an informed decision whether or not to iitiate insulin | clinic managers, health care providers, and patients |
| 1. Trivedi et al., 2009, USA [55] 2. 70% | 1. qualitative 2. informal qualitative interviews during field-testing | 15 study clinicians across five sites, field-testing consisted of over 300 outpatient visits by 168 patients | examine feasibility and effectiveness of implementation (pilot testing) | mental health care | computerized decision support system for depression (CDSS-D) | study clinicians |
| 1. Urowitz et al., 2012, Canada [49] 2. 80% | 1. mixed methods 2. questionnaire (n=64), telephone interview (n=17) | 17 patients and  64 health care providers | pilot program | Diabetes (T1DM + T2DM) | patient portal: online diabetes management portal | patients, health care providers |
| 1. Varonen et al., 2008, Finland [50] 2. 90% | 1. qualitative 2. Focus group interview (n=39) | 39 physicians in 7 focus groups | planning a generic just-in-time CDSS in the EBMeDS project | general | computerized decision support systems (CDSSs) | physicians |
| 1. Wade-Vuturo et al., 2013, USA [75] 2. 100% | 1. mixed methods 2. focus group interview (n=39), survey (n=54) | 54 Adults with T2DM who had used a patient portal | evaluation of intervention | Diabetes (T2DM) | secure messaging (SM) within a patient portal: MyHealthAtVanderbilt (MHAV) patient portal | patients |
| 1. Wan et al., 2012, Australia [51] 2. 80% | 1. qualitative 2. Semi-structured telephone interviews (n=17) | 22 general practitioners and 2 practice nurses | evaluation of intervention | Diabetes (T2DM) | electronic decision support (EDS) | health care providers |
| 1. Wang et al., 2013, USA [52] 2. 90% | 1. qualitative 2. focus group interview (n=8) | 8 diabetes educators | evaluation of intervention | Diabetes | chronicle diabetes: a data management system that provides tools for diabetes educators to document, track, and report on their patients’ education process | diabetes educators |
| 1. Wildeboer et al., 2018, The Netherlands [76] 2. 100% | 1. mixed methods 2. interviews (n=17), quanitative analyses with patient-reported data (n=209), aggregated at practice level (n=17) | 17 practice staffs | part of randomized controlled trial | Diabetes | decision aids(DA): presented personalized information on risks and treatment for multiple risk factors, treatment effects on complication, etc. | practice staff, patients |
| 1. Yu et al., 2019, Canada [53] 2. 80% | 1. qualitative 2. individual interviews (n=17), usability testing (n=11) | 7 patients, 10 clinicians, 11 patient-clinician dyads | development and refinement of intervention | Diabetes (T1DM or T2DM and two other comorbidities) | shared decision-making (SDM) : MyDiabetesPlan, help prioritize guideline-based disease management in patients with multiple comorbidities | health care providers, patients |
| 1.Zwaanswijk at el., e 2013, The Netherlands [54] 2. 90% | 1. qualitative 2. Interviews (n=17) | 17 health care providers | evaluation of intervention | acute diseases, diabetes, mental health | n-EPR(national electronic patient record) | health care providers |

**Table S2. Detailed Study characteristics**

| 1. Author, Year, Country  2. Quality Rating | Participant demographics | Setting | 1.CFIR domains and constructs  2. Number of times mentioned as facilitator or barrier |
| --- | --- | --- | --- |
| 1. Abidi et al., 2018, Canada [65] 2.100% | The 7 participants for the focus group study included 3 CDEs and 4 patients. All 3 CDEs were females, and of 4 patients, 3 were females, and 1 was male. Ages for CDEs ranged from 29 to 55 years, and patients were aged between 49 and 64 years. All CDEs worked at DMCs. CDEs had a median of 11 years of experience (range 3-19 years). Patients had diabetes for a median of 13.5 years (range 2-25 years). | Primary care practitioner tool | 1. needs and resources, structural characteristics, available resources, access to knowledge 2. 6 |
| 1. Allain et al., 2017, Malawi [88] 2. 66% | 1466 (59%) subjects were female and 1004 (41%) were male. The median age of patients was 53 years, interquartile range 42-62years. Twenty-four were aged <16 years. The median duration of diabetes for prevalent cases was 5 years, interquartile range 2-10 years. | a diabetes specific EMRs introduced in the diabetes clinic at Queen Elizabeth Central Hospital (a high burden ART site) | 1. complexity, needs and resources, available resources 2. 3 |
| 1. Ancker et al., 2015, US [38] 2. 90% | Half of patients were men and half were women; a third (n=7) were black. Ages ranged from 37-89 (mean 64.1; median 66). About two-thirds (n=15) were not currently married. Just over a third (n=8) used English as a second language. One third (n=7) were covered by Medicare (US public insurance for those over age 65); one third (n=7) by Medicaid (US public insurance for those with low income); and the remainder (n=8) by commercial insurance. | general health care(not a specific program): explore role of PHIM | 1. relative advantage, design quality & packaging, privacy & confidentiality, knowledge & beliefs about innovation, self-efficacy 2. 6 |
| 1. Ancker et al., 2015, USA [39] 2. 90% | Half of patients were men and half were women; a third (n=7) were black. Ages ranged from 37-89 (mean 64.1; median 66). About two-thirds (n=15) were not currently married. Just over a third (n=8) used English as a second language. One third (n=7) were covered by Medicare (US public insurance for those over age 65); one third (n=7) by Medicaid (US public insurance for those with low income); and the remainder (n=8) by commercial insurance. | general health care( not a specific program): explore perspeectives about personal data tracking | 1. evidence strength and quality, relative advantage, compatibility 2. 3 |
| 1. Baudendistel, et al., 2015, Germany [66] 2. 80% | third ofthe participating patients (n=4) were living with their diagnosis for less than 1 year, four patients between 1 and 2 years, and four patients for at least 6 years. The average duration since the diagnosis of participating patients was 1.7 (0.8; 6.7) years. | Regional health care settings | 1. access to knowledge, knowledge and beliefs about the innovation, self-efficacy, other personal attributes 2. 8 |
| 1. Brown et al., 2017, USA [72] 2. 80% | 80% racial/ethnic minorities; (mean age = 36) years | large integrated healthcare delivery system | 1. relative advantage, design quality & Packaging, networks & communications, privacy & confidentiality 2. 5 |
| 1. Conway et al., 2019, Scotland [77] 2. 80% | 63% were male, were representation of all age and socioeconomic groups, 789/1,095 (72%) had T2D, 290/1,095 (27%) had T1D; vast majority identified as "White" | NHS scotland interactive website for people with diabetes and their carers | 1. relative advantage, adaptability, complexity, design quality & packaging, available resources 2. 8 |
| 1. Dehnavi et al., 2021, Iran [67]  2. 100% | 12 neuro-oncologists and thoracic oncologists and 19members of the thoracic and neuro-oncology departments, | EHR used during clinical practice of oncological care providers | 1. design quality and usability 2. 2 |
| 1. Desai et al., 2022, USA [68]  2. 100% | 13 were men, 10 were women, | software used in general practices | 1. relative advantage, available resources, access to knowledge 2. 3 |
| 1. Diaz-Garelli et al., 2021, Malawi [84]  2. 60% | NA | within an EHR system used by primary care clinicians at urban clinics in Indianapolis | 1. adaptability, design quality & packaging, structural characteristics, reflecting & evaluating 2. 4 |
| 1. Dikomitis et al., 2015, UK [40] 2. 90% | most of the physicians were men (n=6; 60 percent. The highest frequency belonged to the age range of 30-39 years for both physicians (n=5; 50 percent) and patients (n=3; 37.5 percent). | endocrine and metabolism research center and in a teaching hospital in Iran | 1. external policy and incentives, available resources, access to knowledge, other personal attributes, planning, key stakeholders 2. 6 |
| 1. Dixon et al., 2013, USA [78] 2. 80% | NA | Memorial Sloan Kettering Cancer Center is deploying a major EHR innovation | 1. relative advantage  2. 1 |
| 1. Fontil et al., 2016, USA [41] 2. 90% | 12 english speakers and 6 spanish speakers | large primary care safety net clinic (adult primary care clinic based in ZSFG) | 1. complexity, networks & communications, available resources, self-efficacy 2. 4 |
| 1. Fuji et al., 2015, USA [56] 2. 70% | average 59 years old (range, 28–80 years), had their initial diagnosis of diabetes 13 years ago (range, 1–38 years), were 61% female (n = 36), and were 71% white/Caucasian (n = 42), and all were at least high school graduates (37% had a bachelor’s degree or higher levels of education) | an internal medicine clinic and an endocrinology clinic | 1. relative advantage, design quality & packaging, needs & resources, compatibility, available resources, privacy & confidentiality, self-efficacy, key stakeholders 2. 11 |
| 1. Grant et al., 2015, USA [79] 2. 60% | mean of 17.8 years (SD: 11.4) of clinical practice experience and 27 (61 %) were female. | primary health care | 1. needs & resources, compatibility, relative priority, organizational incentives  2. 5 |
| 1.Groenhof, et al., 2019, Netherlands [69] 2. 80% | NA | CVRM within the environment of the HER for cardiovascular risk management | 1. relative advantage 2. 1 |
| 1.Hans, et al., 2018, Canada [70] 2. 100% | Participants (n = 12) 50% male, mean age = 56.3 | Primary care provider | 1. adaptability, design quality and usability, structural characteristics, knowledge and beliefs about the innovation 2. 4 |
| 1. Heider et al., 2014, USA [59]  2. 30% | NA | primary health care and community care within a district | 1. cosmopolitanism, relative priority, privacy & confidentiality, champions, executing (2), reflecting & evaluating 2. 7 |
| 1. Hess et al., 2007, USA [42] 2. 80% | 51% male, mean age = 54 | primary health care | 1. relative advantage, design quality & packaging, knowledge & beliefs about innovation, key stakeholders 2. 7 |
| 1. Janssen et al., 2021, Australia [71] 2. 90% | three were undertaken with administrative staff and five were undertaken with clinical staff. Clinical staff represented a range of specialties including radiation oncology, surgery and pathology. | EMR of cancer centre (complex clinical setting) | 1. relative advantage, available resources 2. 3 |
| 1. Jethwani et al., 2012, USA [80] 2. 80% | Mean age was 61 years (range 27–87) ; Mean age of the 20 focus group participants was 58 years (SD, 12.18), ranging from 23–69 years. Seventy percent of focus group participants were male. | community based | 1. relative advantage, complexity, compatibility, key stakeholders, reflecting & evaluating 2. 7 |
| 1.Kabukye et al., 2020, Uganda [87] 2. 66% | About 72% were 40 years or younger, 59% were female, and 75% had worked at the organization for 1–10 years. Eighty-three percent ofrespondents were clinical (oncologists, general doctors, nurses and allied health workers), 89% reported using computers at least on a weekly basis, with 80% rating their computer skills as intermediate to advanced. Fifty-six percent reported experience using an EHR, but only 40.4% reported ever receiving EHR training | Uganda Cancer Institute (UCI), a 100-bed tertiary oncology center in Uganda | 1. relative advantage, cost, peer pressure, external policy and incentives, structural characteristics, implementation climate, available resources, self-efficacy, planning 2. 14 |
| 1. Kooij et al., 2018, Netherland [61] 2. 90% | medical professionals (n=7), managers (n=7), and IT employees (n=7), Mean age was 44.8 years (SD 6.7; range 25-61) and 57% (12/21) were female. We included 6 respondents (6/21, 29%) from UMCs, 9 respondents (9/21, 43%) from teaching hospitals, and 6 (6/21, 29%) from general hospitals. Participants’ work experience varied from 6 years or less (10/21, 48%) to more than 21 years | hospital organizations in the Netherlands | 1. relative advantage, adaptability, design quality and usability, needs and resourcces, external policy and incentives, structural characteristics, networks and communications, culture, implementation climate, readiness to implementation, privacy and confidentiality, knowledge and beliefs about the innovation, other personal attributes 2. 20 |
| 1. Lober et al., 2006, USA [43] 2. 80% | mean age = 69 (range: 49 to 92 years of age), 82% were female, and many had chronic diseases. | community based housing authority (HA) | 1. available resources, self-efficacy, other personal attributes 2. 5 |
| 1. Marchak et al., 2019, USA [44] 2. 80% | NA | pedriatic cancer centers | 1. relative advantage, adaptability, complexity, design quality & packaging, privacy & confidentiality, executing 2. 8 |
| 1. Marcolino et al., 2021, Brazil [73]  2. 100% | All recruited providers serve a racially/ethnically diverse (28% Hispanic, 20% African American, 31% Asian, and 17% Caucasian), poor (approximately one-half are uninsured; 40% live at or below 200% of poverty level) and medically complex population, of whom approximately 11% have CK | Primary care provider (CKD management) | 1. relative priority, available resources 2. 2 |
| 1. Marquard et al., 2013, USA [57] 2. 70% | health care professional: 26% (25/96) were physicians, 46% (44/96) were nurses, and 28% (27/96) , mean age = 33, 66% female | primary health care centers in urban and rural areas | 1. structural characteristics, available resources, access to knowledge, other personal attributes 2. 4 |
| 1. Mayberry et al., 2011, USA [74] 2. 100% | 18 male and 8 females. Age range 40 to 79 | High Risk Diabetes Management Program within multispeciality medical group (primary health care) | 1. evidence strength and quality, adaptability, available resources, privacy & confidentiality, self-efficacy, executing 2. 7 |
| 1.McBride, et al., 2014, US [62] 2. 80% | mean age = 56.9; 65% white, 68% female | primary health care | 1. key stakeholders 2. 1 |
| 1. Osborn et al., 2013, USA [74] 2. 80% | mean age= 56.9, 33% male, 50% female, 47% white | academic medical center | 1. relative advantage, knowledge & beliefs about the innovation, self-efficacy 2. 4 |
| 1. Pemu et al., 2019, USA [82] 2. 80% | mean age = 62 | ambulatory clinics | 1. relative advantage 2. 1 |
| 1. Portz, et al., 2019, US [63] 2. 80% | Participants (N=24) were of a mean age of 78 years and were primarily white women | Kaiser Permanente Colorado's established patient portal | 1. relative advantage, adaptability, complexity, design quality and usability, needs and resources, knowledge and beliefs about the innovation, self-efficacy 2. 9 |
| 1. Pratt, et al., 2021, US [64] 2. 100% | primary care clinics ranged in size from 3 to 25 PCCs based in rural, small town, micropolitan, and metropolitan commuting areas. Eligible participants included PCCs (i.e., family medicine, general internists, physician assistants, and nurse practitioners), clinic and nurse managers, and rooming staff (i.e., RN, LPN, MA) | 22 primary care clinics in Minnesota, North Dakota, and Wisconsin | 1. relative advantage, compatibility, available resources 2. 3 |
| 1. Ralston et al., 2004, USA [45] 2. 100% | aged 43-65 (median 58) years and had had type 2 diabetes for one to 14 (median four) years. Eight were married or had partners, one was single; two had high school education, one college education, and four graduate school education; eight were white and one was African-American | patient homes | 1. relative advantage, knowledge & beliefs about the innovation 2. 3 |
| 1. Ronda et al., 2014, The Netherlands [85] 2. 60% | mean age was 63.9 (SD 12.2) years (nonparticipants: mean 64.5, SD 13.8 years; P=.11) and 826 of 1390 (59.42%) were male (nonparticipant group: 1539/3009, 51.15% male patients; P<.001). | primary health care and community care within a district | 1. relative advantage, needs & resources, knowledge & beliefs about the innovation, self-efficacy, key stakeholders 2. 6 |
| 1. Saleem et al., 2005, USA [58] 2. 70% | 35 intake/triage nurses (registered nurses, licensed practical nurses (LPNs), licensed vocational nurses, and health technicians) during patient intake and 55 providers (physicians, residents, NPs, and physician assistants) with their patient | VA(veterans administration) medical centers | 1. adaptability, design quality & packaging, structural characteristics compatibility, relative priority, available resources, reflecting & evaluating 2. 9 |
| 1. Sarkar et al., 2010, USA [86] 2. 60% | mean age = 59, 28% non-Hispanic White, 14% Latino, 21% AfricanAmerican, 9% Asian, 12% Filipino, and 17% multiracial or other ethnicity | primary health care | 1. self-efficacy 2. 1 |
| 1. Solberg et al., 2017, USA [60] 2. 30% | NA | various health care systems | 1. complexity 2. 1 |
| 1. Tieu et al., 2016, USA [83] 2. 80% | racially and ethnically diverse (76% non-white), and was predominantly African American (36%) and female (68%) | safety net hospital (San Francisco General Hospital) | 1. complexity, self-efficacy, other personal attributes 2. 4 |
| 1. Tieu et al., 2015, USA [46] 2. 80% | Patients were predominantly male (82%, 9/11) and African American (45%, 5/11). All patients had been diagnosed with diabetes and the majority had limited health literacy (73%, 8/11). The majority of caregivers were female (80%, 4/5), African American (60%, 3/5), caregivers of individuals with diabetes (60%, 3/5), and had adequate health literacy (60%, 3/5). | safety net hospital (San Francisco General Hospital) | 1. relative advantage, design quality & packaging, available resources, privacy & confidentiality, knowledge & beliefs about the innovation, self-efficacy, other personal attributes 2. 14 |
| 1. Tong et al., 2020, Malaysia [47] 2. 90% | average 69.6 ± 7.1 years and ranged from 55 to 79 years. The duration of them seeking treatment in the clinic was on average 8.8 ± 5.8 years and ranged from 2 to 20 years. Eleven of them had not started insulin therapy. | primary health care | 1. cost, available resources, other personal attributes, key stakeholders 2. 5 |
| 1. Tong et al., 2020, Malaysia [48] 2. 100% | policymaker: 5; doctor: 9; pharmacist: 6; diabetes educator: 3; pharmacist: 6; patients: 15 / mean age = 43.6 | public health clinic | 1. cost, available resources, leadership engagement, self-efficacy, key stakeholders 2. 8 |
| 1. Trivedi et al., 2009, USA [55] 2. 70% | NA | public mental health clinics | 1. adaptability, structural characteristics, compatibility, available resources, self-efficacy 2. 6 |
| 1. Urowitz et al., 2012, Canada [49] 2. 80% | 8 male, 9 female | primary health care and community care within a district | 1. relative advantage, adaptability, complexity, design quality & packaging, relative priority, available resources  2. 17 |
| 1. Varonen et al., 2008, Finland [50] 2. 90% | mean age = 47 (27-56), 44% female 22 in primary care, 17 in secondary care | primary and secondary health care | 1. evidence strength and quality, relative advantage, adaptability, complexity, available resources, access to knowledge, knowledge & beliefs about the innovation  2. 9 |
| 1. Wade-Vuturo et al., 2013, USA [75] 2. 100% | mean age = 57.1, 65% female, 76% caucasian/white, 20% african american/black | primary care clinic | 1. evidence strength and quality, relative advantage, knowledge & beliefs about the innovation, key stakeholders, executing 2. 8 |
| 1. Wan et al., 2012, Australia [51] 2. 80% | 14 male, 8 female | primary health care | 1. relative advantage, adaptability, design quality & packaging, needs & resources, compatibility, organizational incentives, available resources, knowledge & beliefs about the innovation 2. 12 |
| 1. Wang et al., 2013, USA [52] 2. 90% | all female and white (mean age, 53.75 ± 10.22 years) | diabetes education | 1. relative advantage, complexity, design quality & packaging, external policy & incentives 2. 5 |
| 1. Wildeboer et al., 2018, The Netherlands [76] 2. 100% | 12 specialized assistants, 4 general assistants, 1 diabetes nurses | Dutch PORTDA-diab trial. | 1. relative advantage, adaptability, design quality & packaging, compatibility, other personal attributes  2. 8 |
| 1. Yu et al., 2019, Canada [53] 2. 80% | 7 patients, 6 physicians, 2 nurses, 1 dietitian, 1 pharmacist | family health teams in the academic and community settings | 1. complexity, cosmopolitanism, implementation climate, relative priority, available resources, knowledge & beliefs about the innovation, self-efficacy, other personal attributes, executing 2. 10 |
| 1.Zwaanswijk at el., e 2013, The Netherlands [54] 2. 90% | 12 male, 5 female; 6 acute care, 5 diabetes care, 6 ambulatory mental health care; 7 GP, 2 GP assistant, 2 psychologist, 2 psychiatrist, 2 medical informatics expert, 2 other | acute care, diabetes care, and ambulatory mental health care. | 1. relative advantage, adaptability, complexity, design quality & packaging, structural characteristics, networks & communications, privacy & confidentiality 2. 9 |
