# Supplementary material for: Using an Integrated Framework to Investigate the Facilitators and Barriers of Health Information Technology Implementation in Noncommunicable Disease Management: Systematic Review
Source: J Med Internet Res. 2022 Jul 20;24(7):e37338. doi: 10.2196/37338 (PMC9350822; doi:10.2196/37338)
Supplement: Multimedia Appendix 8 [file jmir_v24i7e37338_app8.docx]

**Multimedia Appendix 8. Descriptive theme definitions and representative quotes**

| Descriptive theme | Definition | Representative quote |
| --- | --- | --- |
| **Characteristics of the intervention** | | |
| Evidence strength and quality | Stakeholders’ perceptions of the quality and validity of evidence supporting the belief that the innovation will have desired outcomes. | **Facilitator:** “reliable knowledge base and that trusted peers are developing the system”[50] **Barrier:** “Providers often perceived patient-recorded data as unreliable. The lack of confidence was attributed to perceived lack of diligence, moral valence of the data (with patients unwilling to “admit” undesirable numbers), and fear of consequences. The most striking example, told by a provider, was a woman who faked her daughter’s blood glucose log to persuade the doctor to delay starting insulin therapy” [39] |
| Relative advantage | **Facilitator:** Stakeholders’ perception of the advantage of implementing the innovation versus an alternative solution. **Barrier:** Stakeholders’ perception of the disadvantage of implementing the innovation versus an alternative solution. | **Facilitator:** “It helped me understand that, so it made me watch my sugar more often when I was in pain. I would check my sugar to find out if it was high or low and try to tie in the highness of the sugar with the pain I was in or you know, stuff like that and with the eyesight as well it took a lot of, like, what I was really worried about was the eyesight when I found I was diabetic and it helped me with that quite a bit...” [49]  **Barrier:** “Occasionally a patient used the portal to report symptoms that should have triggered an office visit. Also, I am concerned they would use this instead of having their regular formal lab evaluation and follow-up visit” [49] |
| Adaptability | The degree to which an innovation can be adapted, tailored, refined, or reinvented to meet local needs. | **Facilitator:** “In order for the software program to be used in all cases in "real world" settings, we found that considerable treatment flexibility was necessary.” [55]  **Barrier:** Two providers established during a patient encounter that a reminder was not applicable because of (1) a previous misdiagnosis for diabetes and (2) a case in which the reminder was ‘‘reminding’’ the provider to reﬁll the patient’s angiotensin-converting enzyme (ACE) inhibitor when in fact a reﬁll order was not needed. In this second case, the system looks for the patient ﬁlling a prescription, not the provider ordering the medication[58] |
| Complexity | **Barrier:** Perceived difficulty of the innovation, reflected by duration, scope, radicalness, disruptiveness, centrality, and intricacy and number of steps required to implement. | Registry creation and file transfer issues had to be resolved by project staff. Multiple steps were often required to run and upload the registries.[59] |
| Design Quality and Usability | Perceived excellence in how the innovation is bundled, presented, and assembled.  **Facilitator**: perceived excellence on design, visualization, and system function  **Barrier:** problems related to data quality, design, and system function | **Facilitator:** “It’s nice to give a visual thing for the actual patient themselves. It makes them feel included when you share that information with them and because it comes from their file also they think that it makes it seem a little bit more personal. Print it out into, onto the particular specified screen that you like. So say for example they’re not to increase their activity then you can expand and go into that and then you can print out information so you can take it step by step”[51]  **Barrier:** “I think it takes a bit longer because it’s you know a lot of ticking and putting things in – it doesn’t always automatically do it for some reason.”[51] |
| Cost | **Barrier:** Costs of the innovation and costs associated with implementing the innovation including investment, supply, and opportunity costs. | “I can tell you for general public, they will not pay, even RM1.50 (USD 0.35).” |
| **Outer setting** | | |
| Needs and Resources* | **Facilitator:** desire or needs of stakeholders  **Barrier:** lack of desire or needs of stakeholders | **Facilitator:** “There should be apps that talk more about things like...distress, depression and psychology...I mean diabetes is hard, sometimes we are distressed and we need information and we rely a lot on Internet...”[65] **Barrier:** “My A1c has been steady. So I didn’t feel like I really needed to use it as often.that wasn’t particularly useful for me.’’[56] |
| Cosmopolitanism* | **Facilitator:** The degree to which an organization is networked with other external organizations. Includes influences from networks and communications with outgroup (e.g. peer group) | The positive experience with both of these functions among the early adopters had a positive influence on others, via professional and social networks.[59] |
| Peer Pressure | **Facilitator:** Mimetic or competitive pressure to implement an innovation, typically because most or other key peer or competing organizations have already implemented or are in a bid for a competitive edge. | “EHR is strongly recommended and encouraged in many facilities; in fact most private facilities have implemented it”[87] |
| External Policy and Incentives * | **Facilitator, Barrier:** External strategies including policy and regulations (governmental or other central entity), external mandates, recommendations and guidelines, pay-for-performance, collaboratives, and public or benchmark reporting that influence implementation. | **Facilitator:** “Facilitating laws and regulations can be beneficial, and especially the support by the government in the Netherlands for portal implementation is seen as a facilitator.”[61] **Barrier:** “Complying with regulations of the Health Insurance Portability and Accountability Act and maintaining privacy when e-mailing or downloading the snapshot or patient education summary could be problematic” [52] |
| **Inner setting** | | |
| Structural Characteristics | **Barrier:** The social architecture, age, maturity, and size of an organization. | In fact, responsibilities for resolving particular reminders varied not only between the four sites, but also in some cases varied between each speciﬁc clinic within a site. Even when roles were clariﬁed, the assigned responsibility of individual reminders sometimes changed over time, producing confusion.[58] |
| Networks and communications | The nature and quality of webs of social networks, and the nature and quality of formal and informal communications within an organization. **Facilitator:** high-quality and trustworthy networks and communication **Barrier:** lack of conceived trust and communication | **Facilitator:** “Participants perceived their physician as trustworthy and concerned about their health, and noted that they were more likely to read a tailored message that came directly from that individual.”[72] **Barrier:** I’m not feeling any connection with the people that are in there. There’s no camaraderie in seeing names in a chatroom.[41] |
| Culture | **Facilitator:** Norms, values, and basic assumptions of a given organization. | **Facilitator:** “An innovation-oriented culture can help for the reason that the implementation is supported by the organization, the staff are stimulated and feel motivated, and there is a positive mood.”[61] |
| Implementation Climate | The absorptive capacity for change, shared receptivity of involved individuals to an innovation, and the extent to which use of that innovation will be rewarded, supported, and expected within their organization. | |
| -tension for change | **Barrier:** The degree to which stakeholders do not want change in the current situation | **Barrier:** "some senior staffwho would support the implementation ofthe EHR change still have negative attitude towards the need for change. Also, I think people have fear that they may lose their jobs if they implement EHR"[87] |
| -Compatibility | The degree of tangible fit between meaning and values attached to the innovation by involved individuals, how those align with individuals’ own norms, values, and perceived risks and needs, and how the innovation fits with existing workflows and systems. | **Facilitator:** This integration of the clinical topic addressed by the reminder into the template aligns it more directly into the nurse’s workﬂow, thus facilitating its effective use.[58] **Barrier:** Data tracking sometime was felt to conflict with the work of everyday living forcing trade-offs when patients did not have sufficient time or emotional resources. A diabetes patient who had given up self-monitoring of blood glucose said, “It’s too cumbersome for me”. A patient with heart disease who kept a diet log gave it up after it became “overwhelming”.[39] |
| -Relative Priority | **Barrier:** Individuals’ shared perception of the importance of the implementation within the organization. | “I’ve got other things that are pressing on my mind that I've taken, you know, precedence and overridden everything else that's going on and until those matters get taken care of I've put a lot of stuff that I shouldn't, especially the diabetes and that on the back burner until the other stuff gets taken care of…”[49] |
| -Organizational Incentives | **Barrier:** Extrinsic incentives such as goal-sharing, awards, performance reviews, promotions, and raises in salary, and less tangible incentives such as increased stature or respect. | Most intervention group PCPs (80 %) reported barriers to using the Medication Metronome tool including poor alignment with current visit-based reimbursement practices. [79] |
| Readiness to Implementation | Tangible and immediate indicators of organizational commitment to its decision to implement an innovation. | |
| -Leadership engagement | **Barrier:** Commitment, involvement, and accountability of leaders and managers with the implementation of the innovation. | The Clinic B’s manager also voiced that effective implementation ofthe insulin PDA would depend on the diabetes MO in-charge ofdiabetes in the clinic. However, during the researcher’s (WTT) interview with the diabetes MO in-charge, the individual demonstrated a lack ofinterest with the insulin PDA and was also in a hurry to end the interview session.[48] |
| -Available Resources | The level of resources organizational dedicated for implementation and on-going operations including physical space and time. | **Facilitator:** We found that technical support to maintain the server and network was crucial to smooth operation and interfacing speed. [55] **Barrier: “**We don’t have money to print. Our budget has been cut down 20%. Our funding comes from the state health office. Our priority is on drugs but even now we have been cutting down on some non-essentials drugs that we are not giving to patients anymore. Money is an issue”.[48] |
| -Access to Knowledge | **Facilitator:** Ease of access to digestible information and knowledge about the innovation and how to incorporate it into work tasks. Includes statements related to training and education. | It (the WebEx) was really long and drawn out is the honest answer; I think it was half an hour or an hour, I can’t remember. Yes, but actually there was a good eight minute slot that was brilliant that just explained it all, so I would be tempted I think from watching that thing it made a big, eh, it was really useful’.[40] |
| Privacy and Confidentiality* | Perceived appropriateness of measures on  maintaining the security and confidentiality of patient records | “Regardless of what a person says that this site is secured and all that, I just don’t believe it...It’s not only hospitals but pharmaceutical and every researcher will tap into my information. That’s the thing that I worry about.” [46] |
| **Characteristics of individual** | | |
| Knowledge and Beliefs about the innovation | Individuals’ attitudes toward and value placed on the innovation, as well as familiarity with facts, truths, and principles related to the innovation. | **Facilitator:** From a practical perspective, they also noted that a patient with the appropriate knowledge base was an essential requirement for SDM[53] **Barrier:** 4 of 7 nonusers reported they were interested in using the portal, but either had never heard about it, or had heard about it but did not know what its capabilities were.[81] |
| Self-efficacy | Individual belief in their own capabilities to execute courses of action to achieve implementation goals. | **Facilitator:** “My doctor knows that I’m into computers. I’m a major in computers so (using the portal] is up my alley.”[46]  **Barrier:** “I don’t feel comfortable doing it on the computer. I’m not the writer. I don’t spell it right, my sentences... I have to get my dictionary out.”[41] |
| Other personal attributes | A broad construct to include other personal traits such as tolerance of ambiguity, intellectual ability, motivation, values, competence, capacity, and learning style. | **Facilitator:** “I think it also depends on whether the physician is friendly. For those who are quite sympathetic, I think people would take it up. I think to sympathize with people will be half battle won because then they will trust you.”[47] **Barrier:** “We have a lot of Indian patients so some ofthem might not be able to read the PDA in the Malay language. Even if we have a Tamil version, I don’t think the doctors here can read it if the patients write it (in Tamil)”.[48] |
| **Process** | | |
| Planning | The degree to which a scheme or method of behavior and tasks for implementing an innovation are developed in advance, and the quality of those schemes or methods | ““It will require a careful, coordinated roll out . . . over months to years. . .”[87] |
| Engaging | Attracting and involving appropriate individuals in the implementation and use of the innovation through a combined strategy of social marketing, education, role modeling, training, and other similar activities. | |
| -champions | **Facilitator:** Individuals who dedicate themselves to supporting, marketing, and ‘driving through’ an implementation, overcoming indifference or resistance that the innovation may provoke in an organization. | A critical step was to identify and nurture an information technology champion.[59] |
| -key stakeholders | Individuals from within the organization that are directly impacted by the innovation, e.g., physicians, patients, family members | **Facilitator:** Family members increased participants’ usage of a PWP and HIT by facilitating initial access and continued use. “My daughter showed to me in my doctor’s ofﬁce, on the computer in the waiting room. No one in the doctor’s ofﬁce ever approached me about it. If it wasn’t for my daughter, I wouldn’t be a PWP user.”[74]  **Barrier:** The second predictor of patient engagement was found to be clinician (e.g., physicians, nurses, educators, and health coaches) engagement. The number of times a clinician logged in to the system to check on their patients correlated directly with clinical outcomes in their patients. An overwhelming majority of patients stopped using the system because they felt that their clinicians never reviewed their data. [80] |
| Executing | Carrying out or accomplishing the implementation according to plan. | **Facilitator:** It was necessary to work closely with vendors to develop data extraction methodology. Conducting a pilot test with one major vendor helped the project gain momentum. [59]  **Barrier:** The Connection Center application failed to link two patients’ BP monitors to their HealthVault accounts. The Microsoft HealthVault team determined that the BP monitor manufacturer had moved the location of their device drivers, so the pointers in Connection Center were pointing to the wrong driver location.[57] |
| Reflecting & evaluating | **Facilitator:** Quantitative and qualitative feedback about the progress and quality of implementation accompanied with regular personal and team debriefing about progress and experience. | ‘‘Part of the success of CRs is attributed to the feedback tool.. At times, changes are made to the CRs based on this feedback.’’[58] |

Notes: * marked categories are the definitions that was modified from the original CFIR construct codebook to match the context of this study. The domain “Privacy and Confidentiality” has been newly added to the original CFIR constructs.
