# Supplementary material for: Using an Integrated Framework to Investigate the Facilitators and Barriers of Health Information Technology Implementation in Noncommunicable Disease Management: Systematic Review
Source: J Med Internet Res. 2022 Jul 20;24(7):e37338. doi: 10.2196/37338 (PMC9350822; doi:10.2196/37338)
Supplement: Multimedia Appendix 9 [file jmir_v24i7e37338_app9.docx]

**Barriers and Facilitators to the Implementation of HIT by the Integrated Framework**

|  | Barriers | Facilitators | System level |
| --- | --- | --- | --- |
| **Outer Setting** | | | |
| Needs and Resources | Lack of desire[63, 66, 79, 85], lack of need[56, 88] | Need for management and information[65], Self-motivation[65, 66] | Individual (patient) |
|  | Lack of desire[79], lack of need[51] | Motivation to change[61] | Individual (HCP) |
| Cosmopolitanism | None reported | Positive experience of early adopters[53, 59] | Interpersonal (HCP) |
| Peer Pressure |  | Peer pressure[87] | Organizational |
| External Policy and Incentives | Regulation concerns [52, 61], government policies[87], lack of health system support[67] | Laws and regulations [61] | Political |
| **Inner Setting** | | | |
| Structural Characteristics | organizational issues[55, 61, 73, 78], unclear responsibility[58, 65, 70, 90, 103], organizational conflicts[87] | None reported | Organizational |
| Networks and communications | Lack of connection with peers[41], Lack of trust[90] | Trusted relationship [72], communication[61] | Interpersonal |
| Culture | None reported | Innovation-oriented culture[61] | Organizational |
| Implementation Climate | | | |
| -tension for change | Tension for change[87] |  | Organizational |
| -Compatibility | Feels like work[39, 56, 80] | Match workflow[64] | Individual(patient) |
|  | Lack of fit with existing workflow[51, 55, 79] | Integration into workflow[58, 61, 76] | Organizational |
| -Relative Priority | Competing priorities[49, 53] | None reported | Individual(patient) |
|  | Competing priorities[58, 62, 79] | None reported | Individual (HCP) |
|  | Competing priorities[59, 62, 87] | None reported | Organizational (vendors) |
| -Organizational Incentives | Lack of reimbursement[51, 79] | None reported | Organizational |
| Readiness to Implementation | | | |
| -Leadership engagement | Lack of leadership engagement[48] |  | Organizational |
| -Available Resources | Lack of administrative support[77], lack of infrastructure and equipment[40, 48, 57, 64, 87, 88], lack of financial resources[48, 61, 87], lack of workforce[48, 61, 87], increased workload [62, 65, 71] | Administrative support[55, 61], adequate infrastructure[57, 58], adequate financial resources[50], technical support[55, 65] | Organizational |
|  | Insufficient computer or internet[46, 49, 56, 61, 67], lack of financial resources[56] | Conducive environment[47], | Individual (patient) |
|  | Lack of time[48, 49, 51, 53, 58, 61, 73], | None reported | Individual (HCP) |
|  | Lack of assistance[41, 43, 49] | None reported | Interpersonal |
| -Access to Knowledge |  | Training[40, 50, 73] | Individual (HCP) |
|  | Lack of training[67] | Patient education[65, 66] | Individual (patient) |
| Privacy and confidentiality* | Privacy concern[38, 44, 46, 56, 68, 72] | None reported | Individual(patient) |
|  | Privacy concern[59, 90] | None reported | Individual (HCP) |
|  |  | Adequate management of data[44, 57, 72] | Organizational |
|  | Privacy regulations[61] |  | Political |
| **Characteristics of Individual** | | | |
| Knowledge and Beliefs about the innovation | Concerns on diminishing interaction with HCP[46], high expectations[45, 70], lack of knowledge [42, 81, 85], preconceived beliefs[38, 75, 81] | Adequate knowledge base[38, 53] | Individual (patient) |
|  | lake of knowledge[51, 61], past negative experience[50, 63], negative attitude[61], resistance toward change[50, 63], concerns on patient’s role[66] | Positive attitude[61] | Individual (HCP) |
| Self-efficacy | Health literacy [38, 43, 46, 48, 53, 83, 86], lack of digital (computer, internet) skills[41, 43, 46, 56, 57, 63, 66, 81, 83, 85] | Adequate health literacy[46] | Individual (patient) |
|  | lack of digital (computer, internet) skills[55, 87] | None reported | Individual (HCP, staff) |
| Other personal attributes | Cognitive impairment[43], financial status[67], literacy[46, 61, 66, 83], passive attitude[76], physical impairment[43], inadequate knowledge of own health[53, 66] | None reported | Individual (patient) |
|  | Older age[66, 73], Poor communication style[47] | Good communication style[47] | Individual (HCP) |
| **Process** | | | |
| Planning | lack of long-term plans[67] | Strategic implementation process[87] | Organizational |
| Engaging | | | |
| -champions | None reported | identify and nurture champion[59] | Organizational |
| -key stakeholders | Lack of HCP engagement[42, 80] | HCP engagement[80] | Individual (HCP) |
|  | Lack of patient-provider engagement[56] | Physician’s suggestion[47, 48, 67, 75, 85], family support[74] | Interpersonal |
|  | Lack of organizational commitment[53] | None reported | Organizational |
| Executing | Lack of cooperation[57] | Cooperation[44, 53, 59], Patient-provider communication[53, 75], use pre-existing relationships[59] | Interpersonal |
| Reflecting and evaluating | None reported | Feedback from provider[80] | Interpersonal |
|  | None reported | Feedback[58, 59], regular monitoring[78] | Organizational |
